# Comparative analysis of double-stranded RNA degradation and processing in insects

**DOI:** 10.1038/s41598-017-17134-2

**Published:** 2017-12-06

**Authors:** Indrakant K. Singh, Satnam Singh, Kanakachari Mogilicherla, Jayendra Nath Shukla, Subba Reddy Palli

**Affiliations:** 1 Department of Entomology, College of Agriculture, Food and Environment, Agriculture Science Center North, University of Kentucky, Lexington, KY USA; 2 Present Address: Molecular Biology Research Lab., Department of Zoology, Deshbandhu College, University of Delhi, New Delhi, India; 3 Present Address: Punjab Agricultural University, Regional Station, Faridkot, Punjab India; 4 Present Address: Department of Biotechnology, School of Life Sciences, Central University of Rajasthan, Kishangarh, Ajmer, Rajasthan India

## Abstract

RNA interference (RNAi) based methods are being developed for pest management. A few products for control of coleopteran pests are expected to be commercialized soon. However, variability in RNAi efficiency among insects is preventing the widespread use of this technology. In this study, we conducted research to identify reasons for variability in RNAi efficiency among thirty-seven (37) insects belonging to five orders. Studies on double-stranded RNA (dsRNA) degradation by dsRNases and processing of labeled dsRNA to siRNA showed that both dsRNA degradation and processing are variable among insects belonging to different orders as well as among different insect species within the same order. We identified homologs of key RNAi genes in the genomes of some of these insects and studied their domain architecture. These data suggest that dsRNA digestion by dsRNases and its processing to siRNAs in the cells are among the major factors contributing to differential RNAi efficiency reported among insects.

## Introduction

There is a constant competition between insects and plants for their co-existence in nature. Every year farmers spend billions of dollars in plant protection programs to deal with the control of crop pests. Use of multiple pesticides is still the primary option for the control of these pests. However, insects are continuously developing resistance to the insecticide used for their management resulting in insecticide resistance as an ever-growing, complicated and global problem^1^. Besides, there is a growing concern about the effect of pesticides on non-target insects and other organisms. Genetically modified crops, expressing insecticidal proteins derived from the bacterium, *Bacillus thuringiensis* (Bt)^2^ also provide a good method to control many economically important insect pests. But, this is not a foolproof strategy as all the insect species are not equally sensitive to Bt toxins and insects have started developing resistance against these toxins^3^. Therefore, there is an urgent need to develop sustainable alternative strategies for the control of insect pests. RNA interference (RNAi) could be one of the promising tools for developing target specific insect pest management methods^4,5^.

RNA interference (RNAi) is a posttranscriptional gene silencing mechanism where exogenous double-stranded RNAs (dsRNA) suppress the expression of target genes by triggering degradation of its mRNAs in a cell^6,7^. In the cell, dsRNA molecules are cleaved by an endonuclease, Dicer, into 21–23 bp short interfering RNAs (siRNAs), which are then recruited to a multi-protein RNA-induced silencing complex (RISC) and guided to the complementary mRNA for degradation^8,9^. RNAi had a huge impact on basic research for investigating gene function. Besides, RNAi has been proposed as a potential strategy to help in solving problems in human and plant health as well as in agricultural pest management^10–12^.

RNAi has provided a valuable tool to knockdown the expression of genes in insects for which genetic tools are not readily available^13^. Transgenic corn plants expressing hairpin dsRNA to target the vacuolar ATPase A subunit gene provided significant protection against western corn rootworm (WCR), *Diabrotica virgifera virgifera* ^14^. Injection and feeding are the most widely used approaches for the introduction of *in vitro* synthesized dsRNA in insects which leads to the activation of RNAi pathway. RNAi efficiency varies considerably among insects belonging to different orders^15,16^. Besides, the mode of dsRNA delivery also affects the efficiency of RNAi. RNAi in the German cockroach, *Blattella germanica*, is effective for many genes tested at different life stages both by injection as well as feeding methods^17–20^. In *Aedes aegypti*, dsRNA injection is the most successful methods of delivery^21,22^ whereas, dsRNA feeding has been found to be successful only in few cases^23,24^. In the desert locust, *Schistocerca gregaria*, an orthopteran insect, significant knockdown of target genes was observed after dsRNA injection, but the RNAi response was much less after dsRNA feeding^25^. Further, many caterpillars are not very amenable to RNAi either by injection or feeding dsRNA^16^.

For widespread use of RNAi methods for insect pest management, it is important to characterize and understand the mechanisms of RNAi in different insects. In the current study, we have performed *in silico* identification and characterization of the genes coding for the major components of RNAi machinery including Dicers, Argonauts, R2D2, Sid-1, and dsRNases from insects belonging to major orders and analyzed their domain architecture. We also studied dsRNA degradation and processing using naked and labeled dsRNA, respectively.

## Results

We analyzed the degradation of dsRNA by the dsRNases present in the body fluids (lumen contents and hemolymph) and the processing of dsRNA to siRNA in 37 insects from five orders. Incubation of dsRNA with different concentrations of body fluids and feeding/injection of ^32^P labeled dsRNA were performed. As described below, a wide variation in dsRNA digestion by dsRNases and in dsRNA processing was detected in insects tested.

### Coleoptera

**Figure 1:**
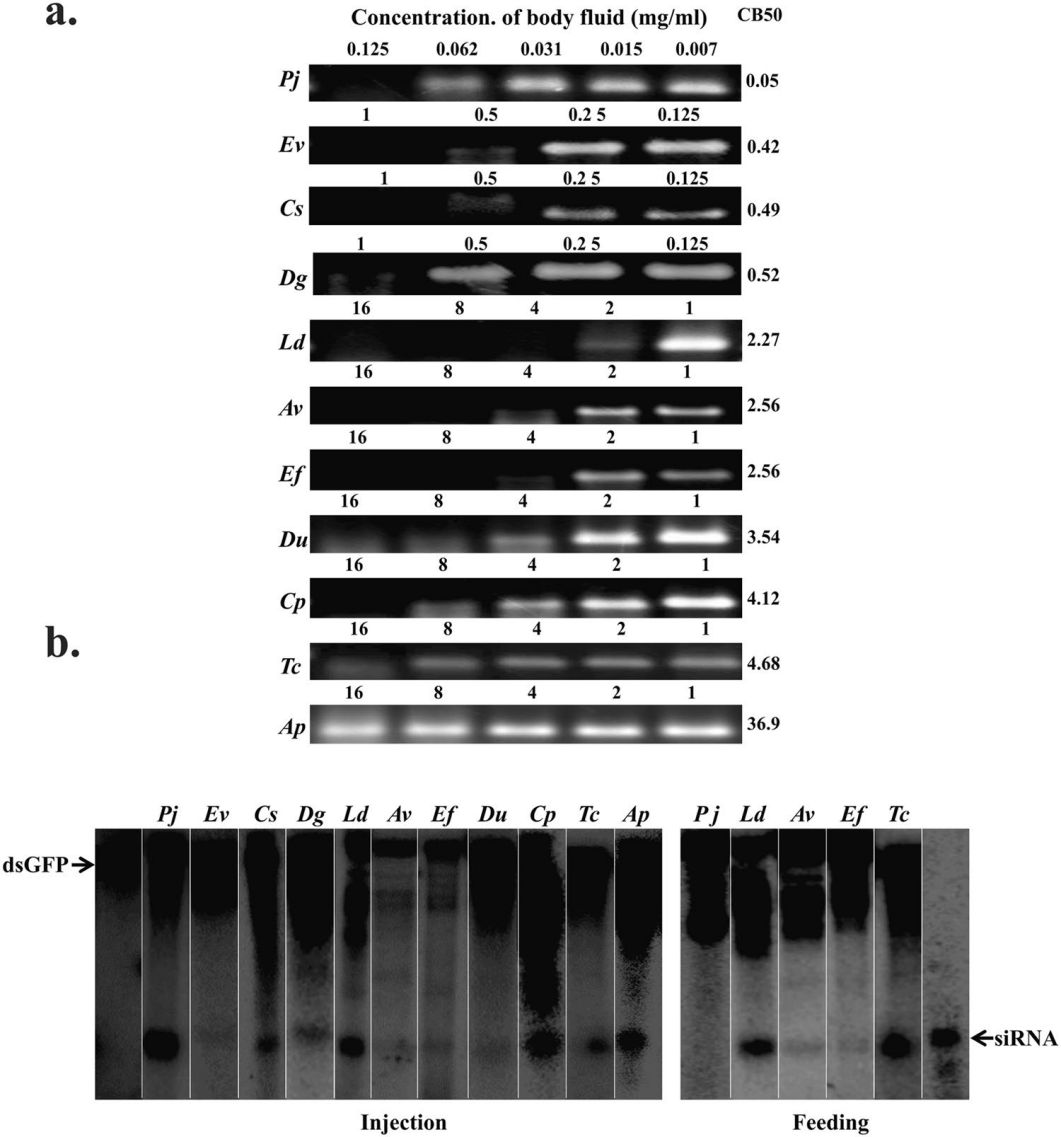
(**a**) dsRNA degradation assay in coleopteran insects: The agarose gels showing dsRNA degradation pattern by body fluids collected from *Popillia japonica*, Pj; *Epilachna varivestis*, Ev; *Coccinella septempunctata*, Cs; *Disonycha glabrata*, Dg; *Leptinotarsa decemlineata*, Ld; *Acalymma vittatum*, Av; *Epitrix fuscula*, Ef; *Diabrotica undecipunctata*, Du; *Chauliognathus pensylvanicus*, Cp; *Tribolium castaneum*, Tc and *Agrilus planipennis*, Ap. 300 ng dsGFP was incubated with 20 μl of serially diluted (0.007 to 16 mg/ml with 1XPBS) body fluid for 1 hr. The samples were run on 1% agarose gel. The relative band intensity was quantified by using ImageJ software. Values are relative to the control, arbitrarily fixed at 100%. The CB50 values were calculated using probit analysis. (see Figure S1a for complete gel images). (**b**) dsRNA processing study in coleopteran insects. Eight million CPM ^32^P labeled dsGFP was injected/fed to *Popillia japonica*, Pj; *Epilachna varivestis*, Ev; *Coccinella septempunctata*, Cs; *Disonycha glabrata*, Dg; *Leptinotarsa decemlineata*, Ld; *Acalymna vittatum*, Av; *Epitrix fuscula*, Ef; *Diabrotica undecipunctata*, Du; *Chauliognathus pensylvanicus*, Cp; *Tribolium castaneum*, Tc and *Agrilus planipennis*, Ap. Total RNA were isolated from these insects at 72 hr after injection/feeding and resolved on 8M urea 16% polyacrylamide gels. The gels were dried and exposed to a phosphor Imager screen, and the image was scanned using a phosphorImager. The lanes labeled dsGFP and siRNA show intact ^32^P labeled dsRNA and 23 nucleotide siRNA respectively. (see Figure S2 for complete gel images).

*Popillia japonica*, *Epilachna varivestis*, *Coccinella septempunctata*, *Disonycha glabrata*, *Leptinotarsa decemlineata*, *Acalymna vittatum*, *Epitrix fuscula*, *Diabrotica undecimpunctata*, *Chauliognathus pensylvanicus*, *Tribolium castaneum* and *Agrilus planipennis* from order Coleoptera were included in the study. The concentration of body fluid required to degrade 50% of dsRNA (CB50) in coleopteran insects varied from 0.05 to 36.86 mg/ml. *P. japonica* and *C. septempunctata* showed higher dsRNA degrading efficiency. Therefore, the CB50 values for these insects are lower when compared to those in other coleopteran insects such as *A. planipennis* where the dsRNA was not degraded completely even at a high concentration of body fluid (16 mg/ml) (Fig. 1a). Coleopteran insects showed an efficient processing of fed or injected dsRNA to siRNA. A band equivalent to the size of siRNA was detected in the total RNA isolated from dsRNA fed or injected coleopteran insects tested except in *P. japonica* fed on dsRNA (Fig. 1b).

### Lepidoptera

**Figure 2:**
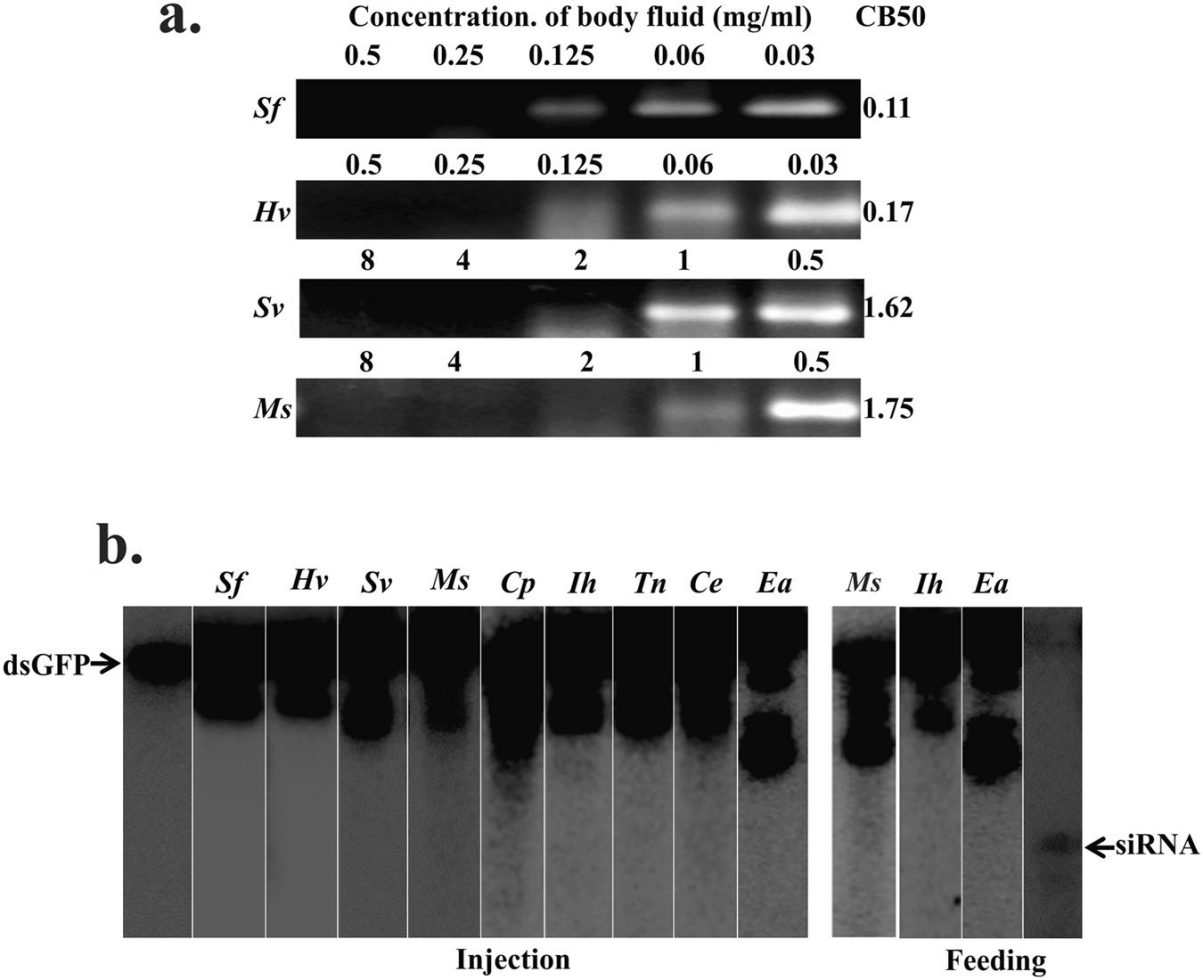
(**a**) dsRNA degradation assay in lepidopteran insects: The dsRNA degradation pattern in *Spodoptera frugiperda*, Sf; *Heliothis virescens*, Hv; *Spilosoma virginica*, Sv; and *Manduca sexta*, Ms; hemolymph was analyzed after 1hr incubation with hemolymph as described in Fig. 1a legend. (see, Figure S1b for complete gel images). (**b**) Processing of dsRNA in Lepidopteran insects. Total RNA isolated from *Spodoptera frugiperda*, Sf; *Heliothis virescens*, Hv; *Spilosoma virginica*, Sv; *Manduca sexta*, Ms; *Cydia pomonella*, Cyp; *Iridopsis humaria*, Ih; *Trichoplusia ni*, Tn; *Colias eurytheme*, Ce, and *Estigmene acrea*, Ea were resolved on urea-acrylamide gels as described in Fig. 1b legend. (see Figure S2 for complete gel images).

*Spodoptera frugiperda*, *Heliothis virescens*, *Spilosoma virginica*, *Manduca sexta*, *Cydia pomonella*, *Iridopsis humaria*, *Trichoplusia ni*, *Colias eurytheme* and *Estigmene acrea* from Lepidoptera were included. A very low concentration of hemolymph from these insects was found to be sufficient to degrade dsRNA within an hour (Fig. 2a). Among the lepidopteran insects tested, *S. frugiperda* hemolymph showed the highest dsRNA degradation activity, and the *M. sexta* hemolymph showed the lowest dsRNA degradation activity. After injection or feeding with labeled dsRNA, no band in the size range of siRNA was detected in the total RNA isolated from lepidopteran insects tested (Fig. 2b).

### Hemiptera

**Figure 3:**
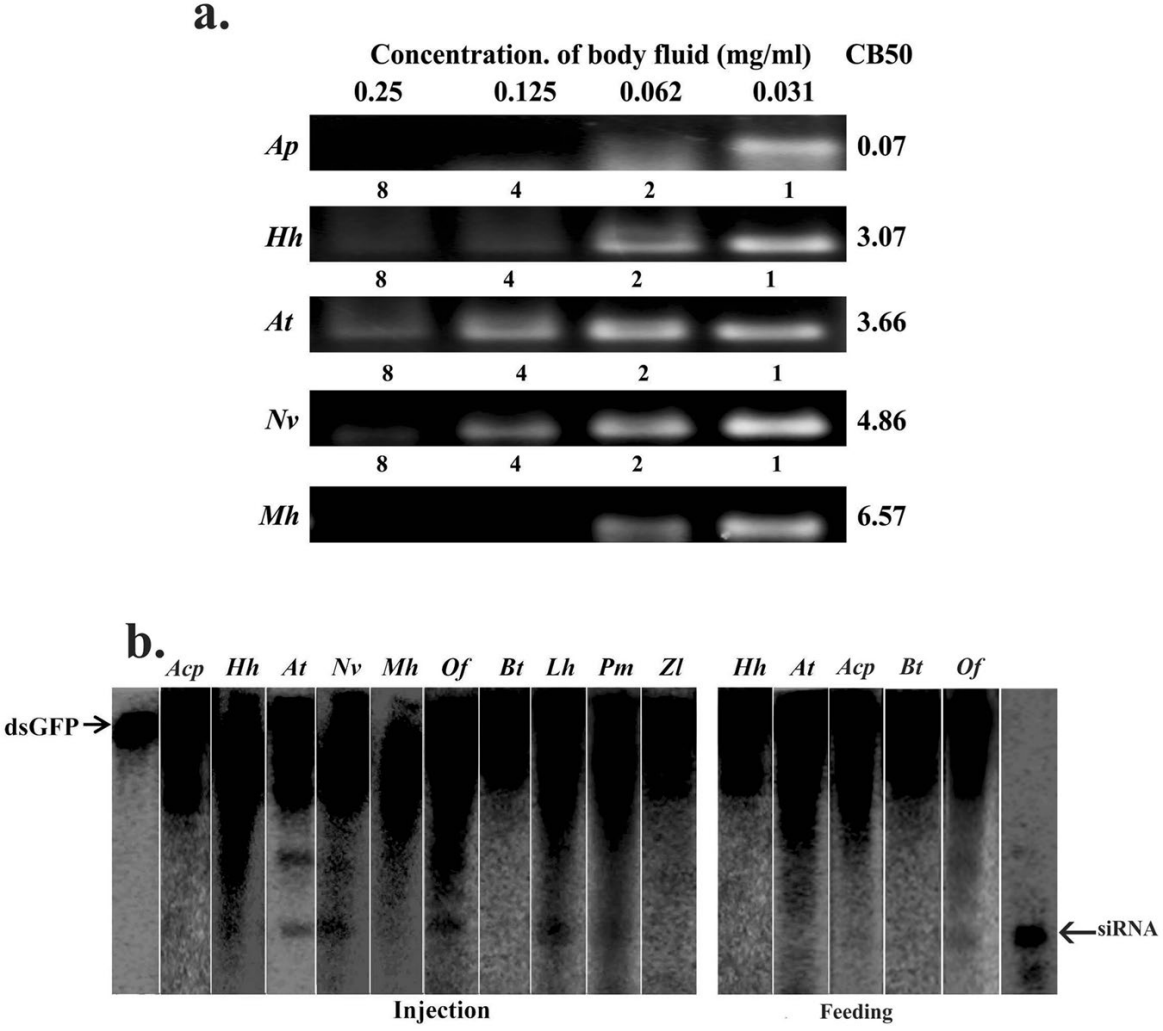
(**a**) dsRNA degradation assay in hemipteran insects: The dsRNA degradation by body fluids collected from *Acyrthosiphon pisum*, Acp; *Halyomorpha halys*, Hh; *Anasa tristis*, At; *Nezara viridula*, Nv, and *Murgantia histrionica*, Mh analyzed as described in Fig. 1a legend. (see Figure S1c for complete gel images). (**b**) Processing of dsRNA in hemipteran insects. Total RNA isolated from *Acyrthosiphon pisum*, Acp; *Halyomorpha halys*, Hh; *Anasa tristis*, At; *Nezara viridula*, Nv; *Murgantia histrionica*, Mh; *Oncopeltus fasciatus*, Of; *Bemisia* tabaci, Bt; *Lygus hesperus*, Lh; *Podisus maculiventris*, Pm; and *Zelus longipes*, Zl were resolved on urea-acrylamide gels as described in Fig. 1b legend. (see Figure S2 for complete gel images).

Ten hemipteran insect species (*Acyrthosiphon pisum, Halyomorpha halys, Anasa tristis, Nezara viridula, Murgantia histrionica, Oncopeltus fasciatus, Bemisia tabaci, Lygus hesperus, Podisus maculiventris and Zelus longipes*) were studied. CB50 value for hemipteran insects ranged between 0.07 mg/ml and 6.56 mg/ml. The lowest value was recorded for *A. pisum*, and the highest value was recorded for *M. histrionica* (Fig. 3a). No siRNA band was detected in the total RNA isolated from the three hemipteran insect species fed on dsRNA (Fig. 3b). Bands equal to the size of siRNA were detected in the total RNA isolated from *A. tristis, N. veridula, O. fasciatus* and *L. hesperus* injected with labeled dsRNA (Fig. 3b). The total RNA isolated from other hemipteran insects showed light siRNA band.

### Diptera

**Figure 4:**
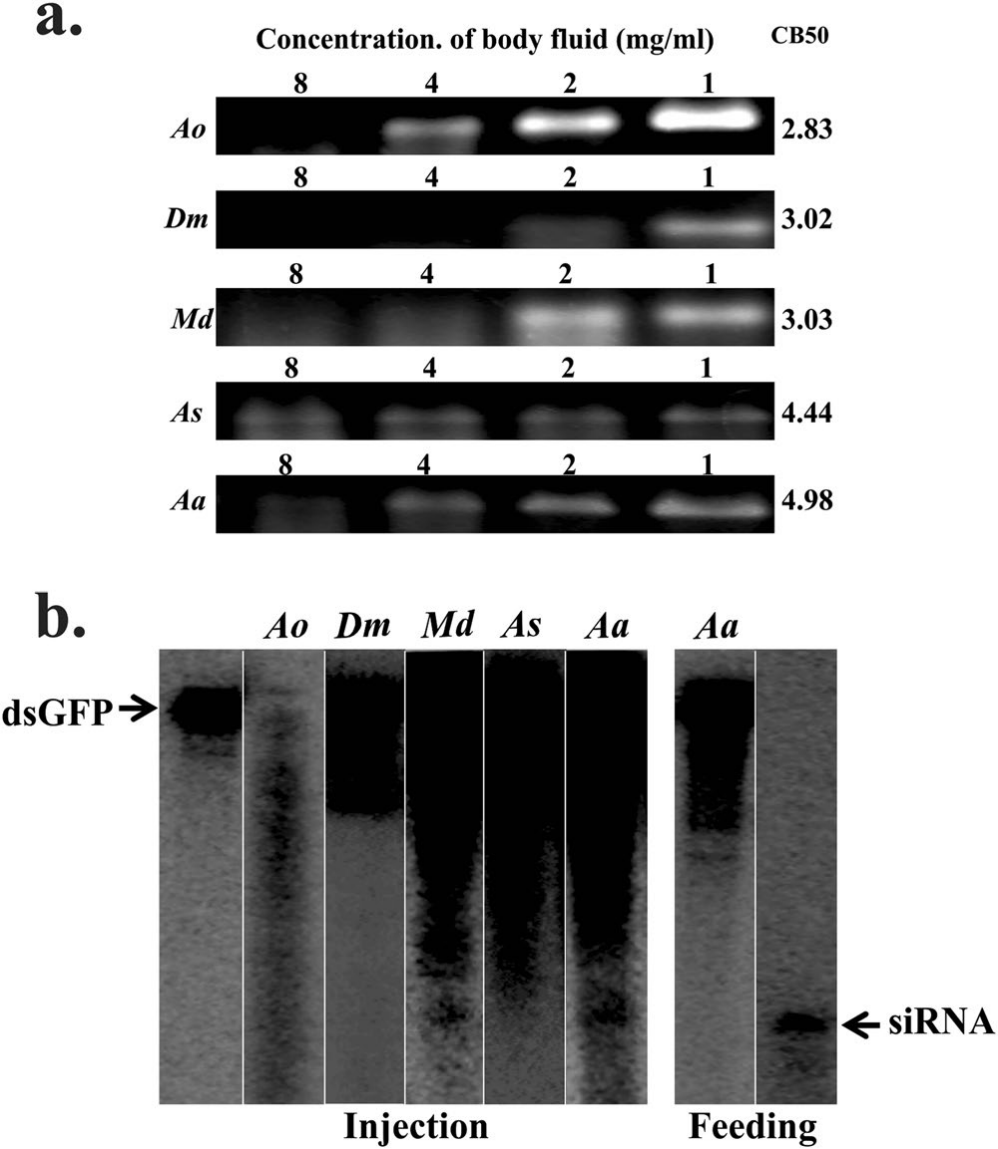
(**a**) dsRNA degradation assay in dipteran insects: The dsRNA degradation by body fluids collected from *Allograpta obliqua*, Ao; *Drosophila melanogaster*, Dm; *Musca domestica*, Md; *Anastrepha suspensa*, As; and *Aedes aegypti*, Aa; were analyzed as described in Fig. 1a legend. (see Figure S1d for complete gel images). (**b**) Processing of dsRNA in dipteran insects. Total RNA isolated from *Allograpta obliqua*, Ao; *Drosophila melanogaster*, Dm; *Musca domestica*, Md; *Anastrepha suspensa*, As; and *Aedes aegypti*, Aa were resolved on urea-acrylamide gels as described in Fig. 1b legend. (see Figure S2 for complete gel images).

*Allograpta obliqua*, *Drosophila melanogaster*, *Musca domestica, Anastrepha suspensa* and *Aedes aegypti* from Diptera were included in the study. Body fluids obtained from all the dipteran insects studied, showed the degradation of dsRNA (Fig. 4a). The CB50 values (2.83 mg/ml to 4.98 mg/ml) for dipterans are in the following order: *A. obliqua* > *D. melanogaster* > *M. domestica* > *A. suspensa* > *A. aegypti*. The siRNA band was detected in the total RNA isolated from *M. domestica* and *A. aegypti* injected with dsRNA (Fig. 4b). No siRNA band was detected in the total RNA isolated from *A. aegypti* fed with dsRNA (Fig. 4b).

### Orthoptera

**Figure 5:**
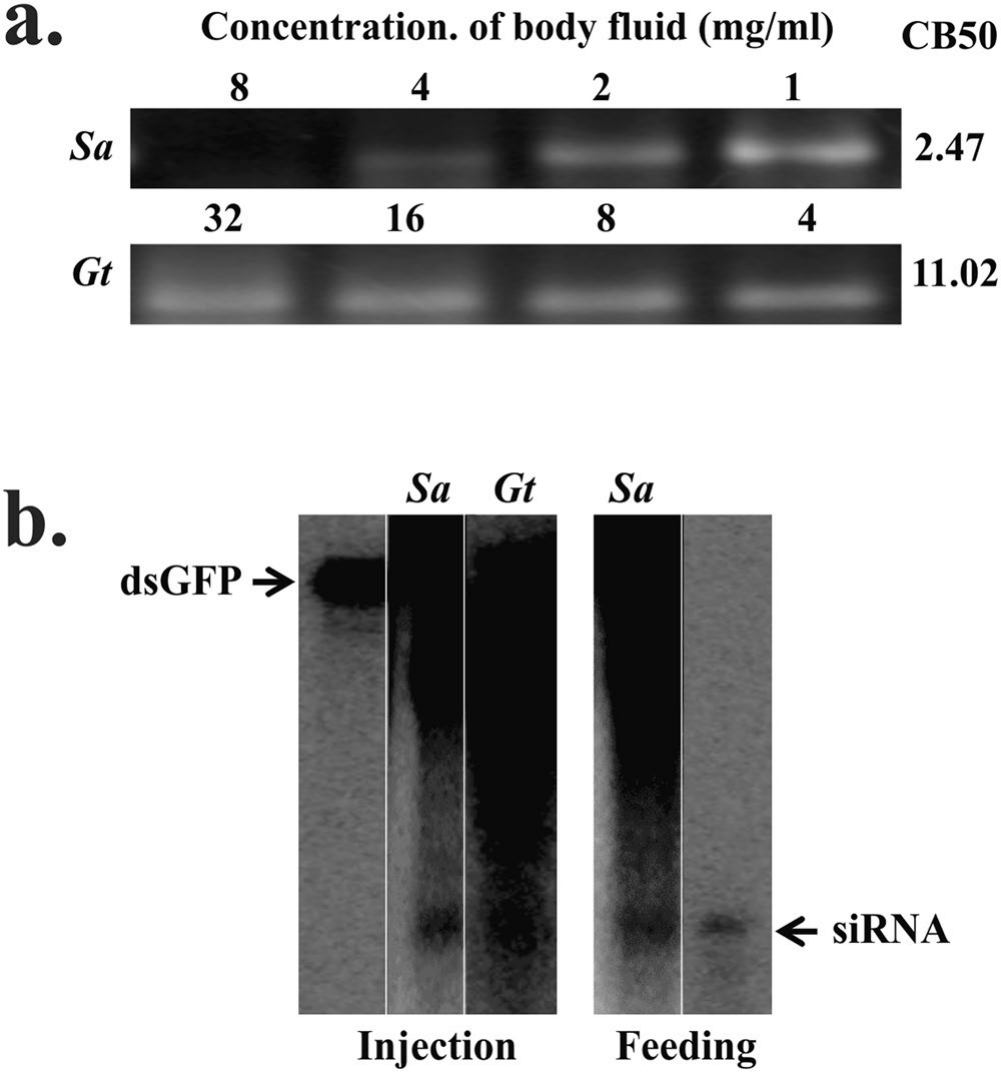
(**a**) dsRNA degradation assay in orthopteran insects. The dsRNA degradation by body fluids collected from *Syrbula admirabilis*, Sa; and *Gryllus texensis*, Gt; were analyzed as described in Fig. 1a legend. (see Figure S1e for complete gel images). (**b**) Processing of dsRNA in orthopteran insects. Total RNA isolated from *Syrbula admirabilis*, Sa; and *Gryllus texensis*, Gt; was resolved on urea-acrylamide gels as described in Fig. 1b legend. (see Figure S2 for complete gel images).

*Syrbula admirabilis* and *Gryllus texensis* from Orthoptera were included. Body fluid collected from *G. texensis* (CB50 2.47 mg/ml) showed higher dsRNase activity than that in *S. admirabilis* (CB50 11.02 mg/ml, Fig. 5a). Both the orthopteran insect species showed processing of injected dsRNA to siRNA. Interestingly, in the case of *S. admirabilis* the dsRNA fed was processed to siRNA (Fig. 5b).

### Characterization of core RNAi machinery genes: Identification of domains

**Figure 6:**
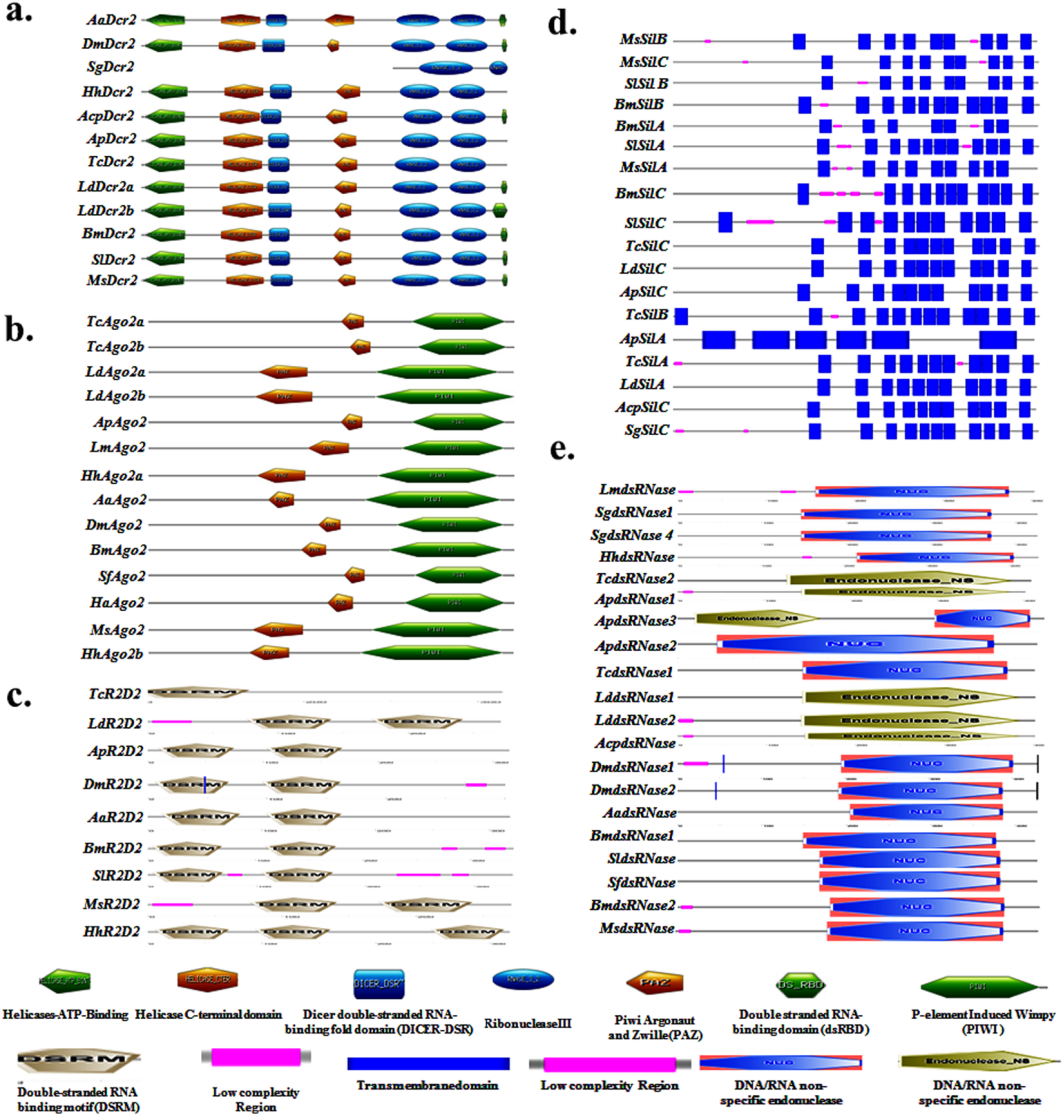
(**a**) Comparison of the domain architecture of Dicer2 (Dcr2) proteins: The domains in Dcr2 proteins were identified by ScanProsite. The species and their accession numbers are: *Aedes aegypti*, Aa, AAW48725.1; *Drosophila melanogaster*, Dm, NP_523778.2; *Schistocerca gregaria*, Sg, AFY13245.1; *Halyomorpha halys*, Hh, XP_014275311.1; *Acyrthosiphon pisum*, Acp, XP_016665093.1; *Agrilus* planipennis, Ap, AJF15703.1; *Tribolium castaneum*, Tc, NP_001107840.1; *Leptinotarsa decemlineata*, Ld, Dcr2a-AKQ00041.1, Dcr2b-AKQ00042.1; *Bombyx mori*, Bm, NP_001180543.1; *Spodoptera litura*, Sl, AHC98017.1; and *Manduca sexta*, Ms, JH668653.1. (**b**) Comparison of domain architecture of Argonaute2 (Ago2) proteins: The domains in Ago2 proteins were identified by ScanProsite. The species included in the above analysis and their accession numbers are: *Tribolium castaneum*, Tc, Ago2a-NP_001107842.1, Ago2b-XP_008192985.1; *Leptinotarsa decemlineata*, Ld, Ago2a-AKQ00044.1, Ago2b-AKQ00045.1; *Agrilus* planipennis, Ap, XP_018319532.1; *Locusta migratoria*, Lm, BAW35368.1; *Halyomorpha halys*, Hh, Ago2a-XP_014271332.1, Ago2b-XP_014287702.1; *Aedes aegypti*, Aa, XP_011493002.1; *Drosophila melanogaster*, Dm, NP_648775.1; *Bombyx mori*, Bm, NP_001036995.2; *Spodoptera litura*, Sl, AHC98010.1; *Helicoverpa armigera*, Ha, ADL27914.1; and *Manduca sexta*, Ms, JH668437.1. (**c**) Comparison of the domain architecture of R2D2 proteins: The domains in R2D2 proteins were identified by SMART. The species included in the above analysis and their accession number are: *Tribolium castaneum*, Tc, A9QW22; *Leptinotarsa decemlineata*, Ld, LDEC002591-PA; *Agrilus planipennis*, Ap, XP_018328507.1; *Drosophila melanogaster*, Dm, NP_609152.1; *Aedes aegypti*, Aa, AJF11544.1; *Bombyx mori*, Bm, NP_001182007.1; *Spodoptera litura*, Sl, AHC98011.1; *Manduca sexta*, Ms, JH668281.1; and *Halyomorpha halys*, Hh, XP_014285641.1. (**d**) Comparison of domain architecture of Sid like protein (Sil) proteins: The domains in Sil proteins were identified by SMART. The species included in and their Sil genes accession numbers are: *Manduca sexta*, Ms, SilA-JH668306.1, SilB -JH668472.1, SilC-JH668472.1; *Spodoptera litura*, Sl, SilA-AHC98014.1, SilB-AHC98013.1, SilC-AHC98015.1; *Bombyx mori*, Bm, SilA-NP_001106735.1, SilB-BAF95807.1, SilC-NP_001106736.1; *Tribolium castaneum*, Tc, SilA-NP_001099012.1, SilB-NP_001103253.1, SilC-NP_001099128.1; *Leptinotarsa decemlineata*, Ld, SilA-ALG36906.1, SilC-ALG36907.1; *Agrilus planipennis*, Ap, SilA-APLA015140-PA, SilC-APLA000678-PA; *Acyrthosiphon pisum*, Acp, SilC-XP_001951907.1; and *Schistocerca gregaria*, Sg, SilC-X2J861. (**e**) Comparison of domain architecture of dsRNase proteins: The domains in dsRNase proteins were identified by SMART. The species and their dsRNase accession numbers: *Locusta migratoria*, Lm, dsRNase-KX652408; *Schistocerca gregaria*, Sg, dsRNase1-AHN55088, dsRNase4-AHN55091; *Halyomorpha halys*, Hh, dsRNase-XP_014282547; *Tribolium castaneum*, Tc, dsRNase1-XP_970494, dsRNase2-XP_015840884; *Agrilus planipennis*, Ap, dsRNase1-XP_018323185, dsRNase2-XP_018334885, dsRNase3-XP_018331412; *Leptinotarsa decemlineata* (Ld) dsRNase1-KX652406, dsRNase2-KX652407; *Acyrthosiphon pisum*, Acp, dsRNase-XP_003242653; *Drosophila melanogaster*, Dm, dsRNase1-AAF49208, dsRNase2-AAM29515; *Aedes aegypti*, Aa, dsRNase-XP_001651912, *Bombyx mori*, Bm, dsRNase1-XP_004922835, dsRNase2-BAF33251; *Spodoptera littoralis*, Sl, dsRNase- CAR92522, *Spodoptera frugiperda*, Sf, dsRNase-CAR92521, and *Manduca sexta*, Ms, dsRNase-JH668361.1.

Bioinformatic analysis was employed to identify and compare domain architecture of core RNAi genes among insects included in the dsRNA degradation and processing studies described above, (the sequence information was obtained from publicly available databases). The Dicer family proteins contained a Helicase ATP BIN, a Helicase CTER, a Dicer double-stranded RNA-binding domain (Dicer-DSR), a PAZ (Piwi/Argonaute/Zwille) domain, two Ribonuclease III and a carboxy-terminal dsRNA-binding domain (dsRBD). Interestingly, the domain architecture of Dicer1 is similar in most insects except in the case of *D. melanogaster*, *A. pisum*, *S. litura* and *B. mori* as the Helicases ATP BIN domain is absent in these insects. Whereas, Ribonuclease III and dsRBD could not be detected in *S. litura* (Fig. S3a; Table 1). Analysis of Dicer2 sequences also showed the presence of signature domains in most of them except in the case of *S. gregaria* where only two Ribonuclease III domains were detected (Fig. 6a; Table 1).

**Table 1:** Protein Domain information: RNAi core machinery genes coding for Dicer, Argonaute, R2D2, dsRNase and Sid family proteins.

	Coleoptera			Lepidoptera					Hemiptera		Diptera		Orthoptera	
	*Ld*	*Tc*	*Ap*	*Sf*	*Sl*	*Ha*	*Ms*	*Bm*	*Ap*	*Hh*	*Dm*	*Aa*	*Lm*	*Sg*
**Dicer1**														
PAZ	+	+	+	−	+	−	+	+	+	+	+	+	+	**−**
Ribonuclease 3	+	+	+	−	−	−	+	+	+	+	+	+	+	**−**
Helicase C	+	+	+	−	+	−	+	+	+	+	+	+	+	**−**
Dicer dimer	+	+	+	−	+	−	+	+	+	+	+	+	+	**−**
DEAD/DEAH	−	−	−	−	−	−	+	−	−	−	−	+	+	**−**
DSRM	+	+	+	−	−	−	−	+	+	+	−	+	−	**−**
**Dicer2/2a/2b**														
PAZ	**+**	**+**	**+**	**−**	**+**	**−**	**+**	**+**	**+**	**+**	**+**	**+**	**−**	**−**
Dicer dimer	**+**	**+**	**+**	**−**	**+**	**−**	**+**	**+**	**+**	**+**	**+**	**+**	**−**	**−**
Ribonuclease 3	**+**	**+**	**+**	**−**	**+**	**−**	**+**	**+**	**+**	**+**	**+**	**+**	**−**	**+**
Helicase C	**+**	**+**	**+**	**−**	**+**	**−**	**+**	**+**	**+**	**+**	**+**	**+**	**−**	**−**
Res III	**+**	**+**	**+**	**−**	**+**	**−**	**+**	**+**	**+**	**+**	**+**	**+**	**−**	**+**
DEAD/DEAH	**+**	**+**	**+**	**−**	**−**	**−**	**−**	**+**	**+**	**−**	**+**	**+**	**−**	**−**
DSRBD/DSRM	**+**	**+**	**−**		**+**		**+**	**+**	**+**		**+**	**+**		
**Argonaute1**														
ArgoL1	**−**	**+**	**+**	**+**	**+**	**−**	**+**	**+**	**+**	**+**	**+**	**+**	**+**	**−**
ArgoL2	**−**	**+**	**+**	**+**	**+**	**−**	**+**	**+**	**−**	**+**	**+**	**+**	**+**	**−**
PIWI	**−**	**+**	**+**	**+**	**+**	**−**	**+**	**+**	**−**	**+**	**+**	**+**	**+**	**−**
ArgMid	**+**	**+**	**+**	**+**	**+**	**−**	**+**	**+**	**−**	**+**	**+**	**+**	**+**	**−**
PAZ	**−**	**+**	**+**	**+**	**+**	**−**	**+**	**+**	**−**	**+**	**+**	**+**	**+**	**−**
ArgoN	**+**	**+**	**+**	**+**	**+**	**−**	**+**	**+**	**+**	**+**	**+**	**+**	**+**	**−**
**Argonaute2/2a/2b**														
PIWI	**+**	**+**	**+**	**+**	**+**	**+**	**+**	**+**	**−**	**+**	**+**	**+**	**+**	**−**
ArgoN	**+**	**+**	**+**	**+**	**+**	**−**	**+**	**+**	**−**	**+**	**+**	**+**	**+**	**−**
ArgoL1	**+**	**+**	**+**	**+**	**+**	**−**	**+**	**+**	**−**	**+**	**+**	**+**	**+**	**−**
ArgoL2	**+**	**+**	**+**	**+**	**+**	**−**	**+**	**+**	**−**	**+**	**+**	**+**	**+**	**−**
ArgoMid	**−**	**+**	**−**	**+**	**−**	**−**	**+**	**−**	**−**	**−**	**+**	**−**	**−**	**−**
PAZ	**−**	**+**	**−**	**+**	**+**	**+**	**+**	**+**	**−**	**+**	**+**	**−**	**+**	**−**
**Argonaute3**														
PIWI	**+**	**+**	**+**	**+**	**−**	**−**	**+**	**+**	**−**	**+**	**+**	**−**	**−**	**−**
ArgoN	**−**	**−**	**+**	**+**	**−**	**−**	**−**	**−**	**−**	**−**	**−**	**−**	**−**	**−**
ArgoL1	**−**	**−**	**+**	**+**	**−**	**−**	**−**	**−**	**−**	**−**	**−**	**−**	**−**	**−**
ArgoL2	**−**	**−**	**+**	**+**	**−**	**−**	**−**	**−**	**−**	**−**	**−**	**−**	**−**	**−**
ArgoMid	**−**	**−**	**+**	**+**	**−**	**−**	**−**	**−**	**−**	**−**	**−**	**−**	**−**	**−**
PAZ	**+**	**+**	**+**	**+**	**−**	**−**	**+**	**+**	**+**	**+**	**+**	**−**	**−**	**−**
**R2D2**														
DSRM	**+**	**+**	**+**	**−**	**+**	**−**	**+**	**+**	**−**	**+**	**+**	**+**	**−**	**−**
**dsRNase**														
Endonuclease NS/S	**+**	**+**	**+**	**+**	**+**	**+**	**+**	**+**	**+**	**+**	**+**	**+**	**+**	**+**
**Sid**														
Sid1	**+**	**+**	**+**	**−**	**+**	**−**	**+**	**+**	**+**	**−**	**−**	**−**	**−**	**+**

*Ld*- *Leptinotarsa decemlineata*; *Tc*- *Tribolium castaneum*; *Ap*- *Agrilus planipennis*; *Sf*- *Spodoptera frugiperda*; *Sl*- *Spodoptera litura*; Ha- *Helicoverpa armigera*; *Ms*- *Manduca sexta*; *Bm*- *Bombyx mori*; *Ap*- *Acyrthosiphon pisum*; *Hh*- *Halyomorpha halys*; *Dm*- *Drosophila melanogaster*; *Aa*- *Aedes aegypti*; *Lm*- *Locusta migratoria*; *Sg*- *Schistocerca gregaria*. [Domain information in the above mentioned tables are based on SCAN PROSITE, SMART, NCBI-CDD and CLC Benchwork with pfam database].

Analysis of Argonaute protein (Ago) family members (Ago1, Ago2/2a/2b, and Ago3) showed the presence of PAZ, ArgoL1, ArgoL2, ArgMid, ArgoN and PIWI (P-element Induced WImpy) domains (Figs 6b, S3b and c; Table 1). Only PAZ and PIWI domains were detected in *A. pisum* and *L. decemlineata* Ago1 proteins, respectively (Fig. 6b and Table 1). The domain architecture analysis of third core protein R2D2 of siRNA pathway revealed that all the insects studied (except *T. castaneum)* from the insect orders, Coleoptera, Lepidoptera, Diptera and Hemiptera, contained duplicate DSRM domains (Fig. 6c and Table 1).

Analysis of putative insect Systemic RNA Interference Deficiency-1 (Sid-1) proteins (Sil A, Sil B and Sil C) showed that these proteins contain tandem repeats of 11 trans-membrane domains separated by extra and intracellular domains (Fig. 6d; Table 1). Low complexity transmembrane regions were detected in Sils from *M. sexta*, *S. litura*, *B. mori*, *T. castaneum* and *S. gregaria*. The proteins coded by putative dsRNase genes studied contain Endonuclease NS (DNA/RNA non-specific endonuclease)/NUC domain and a predicted signal peptide (Fig. 6e and Table 1).

## Discussion

**Figure 7:**
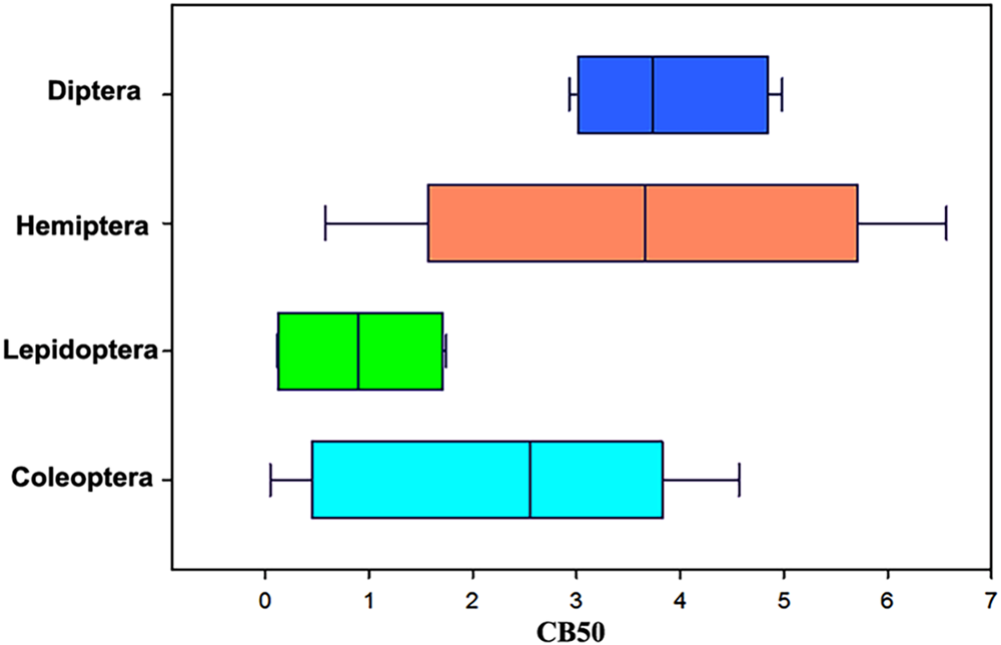
Variability in body fluid/hemolymph concentration required to degrade 50% dsRNA (CB50) in insects from four orders. CB50 values from 25 insects [Diptera (n = 5), Hemiptera (n = 5), Lepidoptera (n = 4) and Coleoptera (n = 11) were included in the analysis].

The efficiency of RNAi has been reported to be variable among insects studied^15,16,26^. In the current study, two different parameters, processing of injected or fed dsRNA to siRNA and degradation of dsRNA by the body fluids, were studied in 37 insect species from five different insect orders (Coleoptera, Lepidoptera, Hemiptera, Diptera, and Orthoptera). A large variability was observed in the dsRNA degradation ability of body fluids collected from insects belonging to these orders (Fig. 7). The CB50 values varied from 0.05 to 36.86 mg/ml. Injected or fed dsRNA was processed into siRNA in all the coleopteran insects tested. Whereas, siRNA band was not detected in total RNA isolated from any of the lepidopterans insects tested. The dsRNA processing efficiency of insects from other orders tested showed the values somewhere between the values in coleopteran and lepidopteran insects. In most of the dipteran and hemipteran insects tested, the injected dsRNA was processed to siRNA, but no siRNA was detected in the total RNA isolated from the insects fed on dsRNA. These data suggest that variability in dsRNA degradation by dsRNases and its processing to siRNA could contribute to variable RNAi efficiency observed in insects.

The efficiency of systemic RNAi response also varies among different insect species. Injection of a small amount of dsRNA induces systemic RNAi response in *C. elegans* and *T. castaneum* ^27,28^ but in lepidopteran insects, a large amount of dsRNA induces only poor RNAi response^29^. Therefore, it is important to study the molecular machinery of RNAi in different insects. Differences in structure and expression of genes coding for proteins involved in RNAi (Dicers, Argonautes, R2D2, Sid-1 like proteins and dsRNases) could affect the efficiency of RNAi. Therefore, in the current study, besides studying the processing and degradation of dsRNA, we also examined the presence of core components of RNAi machinery in different insect species. We retrieved the sequences of core RNAi pathway genes (Dicers, Argonautes, Sid-1 like proteins and dsRNase) of different insects from NCBI, UniProt and i5k databases and analyzed domain architecture of proteins coded by these genes.

Coleopteran insects showed efficient processing of dsRNA into siRNA as evidenced by the presence of a 21–23 bp band in the total RNA isolated from insects injected or fed with labeled dsRNA (Fig. 1b). Our collection of insects included seven coleopteran insects for which RNAi has not been studied previously (See Supplementary Table S1). In addition, a high concentration of body fluid from coleopteran insects is required to degrade dsRNA suggesting that the abundance and/or expression of genes coding for dsRNases is lower in these insects when compared to that in insects from other orders. Several groups^11,14,15,30–34^ reported well-functioning RNAi in coleopteran insects. Duplication of, Argonaute2 in *T. castaneum* ^35–38^ and Argonaute2 and Dicer2 genes in *L. decemlineata* ^39^ suggests that duplication of core RNAi pathway genes in coleopteran insects could also contribute to higher RNAi efficiency observed in these insects. In dsRNA fed *L. decemlineata*, the gene coding for Ago2b is expressed at higher levels as compared to the other core RNAi pathway genes (Dcr2a, Dcr2b and Ago2a)^39^. Some of the technical limitations of the current study including the differences in the efficiency of body fluid collection from insects studied could have contributed to some variability in dsRNA degradation ability of the hemolymph, among insects studied. However, it is likely that the differences in the expression levels and activities of dsRNA degrading enzymes contribute to the variability in dsRNA degradation ability observed among insects studied. Taken together, our data and results from the previous studies suggest that duplication of some genes coding for RNAi machinery proteins, lower levels dsRNases and efficient processing of dsRNA to siRNA are among the major contributors to higher RNAi efficiency in coleopteran insects.

In our study, four lepidopteran insects were tested, and no siRNA was detected in the total RNA isolated from these insects after feeding or injecting dsRNA (Fig. 2b). Also, dsRNA degradation was observed at a lower concentration of hemolymph when compared to that in coleopteran insects. Gene silencing through injection of dsRNA is reported to have variable success in lepidopteran insects^13,16^. Experiments on *C. pomonella* suggests the presence of functional RNAi in this insect as dsRNA mediated knockdown of *cullin-1* gene was observed and, affected the larval growth. Although, no mortality was noticed in this experiment, quantification of *C. pomonella cullin-1* mRNA levels by quantitative reverse-transcriptase real-time PCR revealed a dose-dependent knockdown of the target gene. However, no change in the expression levels of four other genes (*maleless*, *musashi*, *a homeobox* and *pumilio*) tested was observed in the dsRNA mediated knockdown studies^40^. In a few other cases, successful *in-vivo* RNAi experiments have led to important insights into lepidopteran physiology and development. However, in general, RNAi doesn’t appear to be an efficient process in lepidopteran insects. This has stimulated the research on the identification of limiting factors responsible for reduced RNAi efficiency in lepidopteran insects. The entry of dsRNA into cells through the endocytic pathway has been reported^38,41^. In our recent study, we showed entrapment of dsRNA in acidic bodies in the cells from lepidopteran insects, *H. viresences* and *S. furgiperda* ^33^. However, the induction of RNAi was shown in some caterpillars with multiple injections or feeding of higher doses of dsRNA^16,42,43^. In *M. sexta*, dsRNA caused a dose-dependent induction of genes coding for Dcr2 and Ago2, and triggered RNAi^44^. The injection but not feeding of dsGFP in *B. mori* caused an increase in the expression of genes coding for Dcr2 and Ago2^45^. In *B. mori*, overexpression of Ago2 showed both dsRNA and shRNA mediated RNAi response^46^. The midgut lumen contents (pH 11 or 12) collected from lepidopteran insects contain non-specific nucleases which degrade fed dsRNA^43,45,47,48^. Although there are some reports of successful RNAi response in lepidopteran insects after administration of large quantities of dsRNA in general, RNAi response in these insects does not appear to be as efficient as observed in some coleopteran insects. The major contributing factors include degradation by dsRNases and inefficient processing of dsRNA to siRNA.

A variation in the processing of injected/fed dsRNA and dsRNA degradation in the body fluid of hemipteran insects was observed, in the current study. All the tested insects displayed efficient degradation of dsRNA; low concentration of body fluid (CB50 0.07 mg/ml) was sufficient to degrade the dsRNA in the case of the pea aphid (*Acyrthosiphon pisum*) (Fig. 3a). The processing of dsRNA to siRNA was not observed in pea aphid (Fig. 3b) suggesting that dsRNA gets degraded quickly in *A. pisum* before it could be accessed by the RNAi machinery inside the cell. The degradation of dsRNA in the gut or salivary secretions of *L. lineolaris* and *A. pisum* have been reported^49,50^. There are only a few reports of successful RNAi in *A. pisum* ^51–54^. The poor RNAi response observed in hemipteran insects compared to that in coleopteran insects might be due to the salivary secretions causing degradation of dsRNA. Both the salivary secretions and body fluid from aphids were able to degrade the dsRNA, and the administered dsRNA was not able to provoke a response in the expression of the siRNA core machinery genes. The dsRNA-degrading nucleases likely contribute to poor RNAi efficiency in hemipteran insects^52^.

Several previous reports suggest that dipteran (mosquitoes and flies) insects are sensitive to dsRNA or siRNA mediated gene silencing^13,55–57^. The processing of injected dsRNA into siRNA was observed in *A. aegypti* and *M. domestica* but not in *D. melanogaster* (Fig. 4b). A high concentration of the body fluid from all these insects was required for the dsRNA degradation (Fig. 4a). In *D. melanogaster*, dicer1 but not dicer2 is essential for miRISC translational repression^58^. The genomes of *D. melanogaster* and two mosquito species (*Anopheles gambiae* and *A. aegypti*) also do not contain sid-1-like genes^35^.

The injection of dsRNA/siRNA triggers silencing of the target gene in orthopteran insects (locusts and crickets)^59^. Silencing of development and molting related genes (*Lm-TSP* and *Chitin synthase* 1) in locusts induced mortality^60,61^. We detected siRNA bands in the total RNA isolated from dsRNA injected *S. admirabilis* and *G. texensis*. These data support the previous reports on the efficient RNAi upon injection of dsRNA in orthopteran insects (Fig. 5b). The concentrations of hemolymph required to degrade dsRNA varied drastically in these insects; the CB50 value for *S. admirabilis* are lower (2.47 mg/ml) compared to that of *G. texensis* (11.02 mg/ml) (Fig. 5a). Only injection, but not feeding dsRNA caused knockdown of a target gene in case of *S. gregaria* ^25^ and *L. migratoria* ^26^. Expression of non-specific nucleases in the gut of *S. gregaria* was suggested as the reason for inefficient feeding RNAi in *S. gregaria* ^25^. Lower levels of Dcr2 or Ago2 have been suggested as a limiting factor for reduced RNAi observed in the reproductive tissues of *S. gregaria* ^62^. Injection but not feeding dsRNA induces robust RNAi response in *L. migratoria* ^63–65^.

Published reports and the data included in this paper suggest that dsRNA degradation by dsRNases, transport of dsRNA into and within the cells and processing of dsRNA to siRNA are among the major contributing factors for an inefficient RNAi, in insects. Research aimed at uncovering the molecular basis of these mechanisms as well as developing the methods to overcome these limitations should help in improving RNAi efficiency and wide-spread use of this technology in the development of novel methods for controlling pests and disease vectors.

## Methods

### Collection of insects

The insects used in the studies were collected either from the University Farm (University of Kentucky) or laboratory maintained cultures. In the present study, a total of 37 insect species from five orders (Coleoptera, Lepidoptera, Hemiptera, Diptera, and Orthoptera) were tested. The identification of farm-collected insects was done in the Department of Entomology, University of Kentucky, Lexington, USA.

### Collection of body fluid and gel retardation assay

Body fluid was collected from each insect except in the case of lepidopteran larvae, where hemolymph was collected into microcentrifuge tubes containing phenylthiourea dissolved in 1XPBS and kept on ice to prevent melanization. Hemocytes were removed by centrifugation at 13,000 rpm for 10 min at 4 °C. The supernatant was transferred to a new tube and stored at −20 °C for dsRNA degradation assay as described previouly^26,33,43^. Protein concentrations were estimated using Bradford’s assay^66^. Different dilutions of body fluid were prepared based on total protein concentration. The range of serially diluted body fluid used was 0.007 to 16 mg/ml. 300 ng of dsGFP was incubated with the body fluid for 1hr at room temperature. The “concentration of body fluid required to degrade 50% of dsRNA” (CB50) was calculated for different insects using probit analysis^67^. Samples were mixed with loading dye and run on a 1% agarose gels and the gels were stained with ethidium bromide. The dsRNA was visualized with an AlphaImager™ Gel Imaging System (Alpha Innotech, San Leandro, CA) under UV light. The results were analyzed using the Image-J software, and the relative band intensity was calculated as described previously^68^.

### Synthesis of ^32^P UTP labeled and unlabeled dsGFP

^32^P UTP labeled and unlabelled dsGFP were synthesized as described previously^33^. The quality and quantity of dsGFP were checked by agarose gel electrophoresis and NanoDrop-2000 spectrophotometer (Thermo Fisher Scientific Inc., Waltham, MA), respectively. The radioactivity of the labeled dsGFP was measured using a scintillation counter.

### Injection and feeding of ^32^P UTP labeled dsRNA

Insects were starved for 2–3 hr and chilled on ice, dsRNA containing about 8 × 10^6^ counts per minute (CPM) was injected into insect using an insulin syringe. After injections, insects were reared on their respective diets in plastic cups. Total RNA was isolated from these insects. RNA samples were run on 16% polyacrylamide-8M Urea gels. Gels were washed, fixed (10% methanol and ethanol), and dried in a gel drier. Dried gels were exposed overnight to a phosphor-Imager screen, and the screen was scanned in a phosphorImager (Typhoon 9500, GE Healthcare Life Sciences). Details on the methods used for injection and feeding of dsRNA to different insects are included Supplementary Table S2.

### Bioinformatics Analysis

The sequences of RNAi core machinery proteins (Dicers, Argonauts, R2D2, Sil and dsRNase) from different insects were retrieved from NCBI (https://www.ncbi.nlm.nih.gov/), UniProt (http://www.uniprot.org/) and i5k databases (https://i5k.nal.usda.gov/). In some cases, the sequences were obtained by a BLAST search in the i5K workspace @ NAL platform (http://i5k.nal.usd.gov/webapp/blast/) using annotated RNAi core machinery gene sequences of *Tribolium castaneum* as a query. To analyze the domain architecture in the protein sequences of *Dicers* and *Argonauts* genes, Scan-Prosite (http://prosite.expasy.org/scanprosite/), a database of protein families and domains was used^69^. SMART (simple modular architecture research tool) analysis program (http://smart.embl-heidelberg.de/)^70,71^ was used to analyze domain architecture of R2D2, Sid and dsRNase family proteins. The CLC Genomics Workbench and the Pfam database at http://pfam.sanger.ac.uk/ and http://www.clcbio.com/ resources^72^ were also used in the analyses (Table 1).

## Electronic supplementary material

Supplementary Information
