# Supplementary material for: Comparative analysis of double-stranded RNA degradation and processing in insects
Source: Sci Rep. 2017 Dec 6;7:17059. doi: 10.1038/s41598-017-17134-2 (PMC5719073; doi:10.1038/s41598-017-17134-2)

Comparative analysis of double-stranded RNA degradation and processing in insects

Indrakant K. Singh[1,2], Satnam Singh[1,3], Kanakachari Mogilicherla[1], Jayendra Nath Shukla[1,4], & Subba Reddy Palli[1]*

[1]Department of Entomology, College of Agriculture, Food and Environment, Agriculture Science Center North, University of Kentucky, Lexington, KY, USA.

[2]Molecular Biology Research Lab., Department of Zoology, Deshbandhu College, University of Delhi, New Delhi, India.

[3]Punjab Agricultural University, Regional Station, Faridkot-Punjab, India

[4]Department of Biotechnology, School of Life Sciences, Central University of Rajasthan, Ajmer-Rajasthan, India.

*Correspondence addressed to (S.R.P.): rpalli@email.uky.edu

Supplementary Information:

Legends: Supplementary Figures

Supplementary Figure S1a: Complete gel images of dsRNA processing study in coleopteran insects: **A.** *Popillia japonica,* Pj; *Epilachna varivestis*, Ev; *Coccinella septempunctata,* Cs; *Disonycha glabrata,* Dg; *Leptinotarsa decemlineata,* Ld; *Acalymma vittatum*, Av. **B.** *Epitrix fuscula*, Ef; *Diabrotica undecimpunctata*, Du; *Chauliognathus pensylvanicus*, Cp; *Tribolium castaneum*, Tc; and *Agrilus planipennis*, Ap. Selected portion of these gel images have been used in Main Manuscript's Figure 1a. Each gel image lane 1 and 2 are 1kb plus DNA ladder and dsRNA, respectively.

Supplementary Figure S1b: Complete gel images of dsRNA processing study in lepidopteran insects: The agarose gels showing dsRNA degradation pattern from following insects: *Spodoptera frugiperda*, Sf; *Heliothis virescens,* Hv; *Spilosoma virginica,* Sv; and *Manduca sexta*, Ms; Selected portion of these gel images have been used in the Manuscript's Figure 2a. Each gel image lane 1 and 2 are 1kb plus DNA ladder and dsRNA, respectively.

Supplementary Figure S1c: Complete gel images of dsRNA processing study in hemipteran insects: The agarose gels showing dsRNA degradation pattern from following insects: *Acyrthosiphon pisum*, Acp; *Halyomorpha halys*, Hh; *Anasa tristis*, At; *Nezara viridula*, Nv, and *Murgantia histrionica*, Mh; Selected portion of these gel images have been used in the Manuscript's Figure 3a. Each gel image lane 1 and 2 are 1kb plus DNA ladder and dsRNA, respectively.

Supplementary Figure S1d: Complete gel images of dsRNA processing study in dipteran insects: The agarose gels showing dsRNA degradation pattern from following insects: *Allograpta obliqua*, Ao; *Drosophila melanogaster*, Dm; *Musca domestica,* Md; *Anastrepha suspensa*, As; and *Aedes aegypti*, Aa; Selected portion of these gel images have been used in the Manuscript's Figure 4a. Each gel image lane 1 and 2 are 1kb plus DNA ladder and dsRNA, respectively.

Supplementary Figure S1e: Complete gel images of dsRNA processing study in orthopteran insects: The agarose gels showing dsRNA degradation pattern from following insects: from *Syrbula admirabilis*, Sa; and *Gryllus texensis*, Gt; Selected portion of these gel images have been used in the Manuscript's Figure 5a. Each gel image lane 1 and 2 are 1kb plus DNA ladder and dsRNA, respectively.

Supplementary Figure S2 (a-e): Complete gel images of dsRNA processing in insects from different orders. The following insects were selected and highlighted portion of respective gels were used in the manuscript's figure 1b, 2b, 3b, 4b and 5b. *Leptinotarsa decemlineata* (Ld); *Diabrotica undecimpunctata* (Du); *Tribolium castaneum* (Tc); *Agrilus planipennis* (Ap); *Epitrix fuscula* (Ef); *Popillia japonica* (Pj); *Acalymma vittatum* (Av); *Chauliognathus pensylvanicus* (Cp); *Coccinella septempunctata* (Cs); *Epilachna varivestis* (Ev); *Disonycha glabrata* (Dg); *Cydia pomonella* (Cyp); *Manduca sexta* (Ms); *Trichoplusia ni* (Tn); *Spodoptera frugiperda* (Sf); *Heliothis virescens* (Hv); *Spilosoma virginica* (Sv); *Iridopsis humaria* (Ih); *Colias eurytheme* (Ce); *Estigmene acrea* (Ea); *Bemisia tabaci* (Bt); *Acyrthosiphon pisum* (Acp); *Halyomorpha halys* (Hh); *Lygus Hesperus* (Lh); *Oncopeltus fasciatus* (Of); *Podisus maculiventris* (pm); *Zelus longipes* (Zl); *Anasa tristis* (At); *Nezara viridula* (Nv); *Murgantia histrionica* (Mh); *Aedes aegypti* (Aa); *Musca domestica* (Md); *Drosophila melanogaster* (Dm); *Anastrepha suspensa* (As); *Allograpta obliqua* (Ao); *Syrbula admirabilis* (Sa); *Gryllus texensis* (Gt). (^{I}= Injection; ^{F}= Feeding)

Additional insects mentioned in the dsRNA processing gels are: *Cimex lectularius* (Cl); *Reticulitermes flavipes* (Rf); *Folsomia candida* (Fc), *Hypoponera opacior* (Ho); *Tetramorium caespitum* (Ttc); *Panorpa confuse* (Pc); *Periplaneta americana* (Pa).

Supplementary Figure S3a: Comparative domain architecture of (Dicer1) Dcr1 proteins: The Dcr1 proteins predicted by ScanProsite and their Dcr1 accession no. are *Tribolium castaneum* (Tc) (EFA11550.2), *Leptinotarsa decemlineata* (Ld) (LDEC001540-PA), *Agrilus planipennis* (Ap) (XP_018328602.1), *Aedes aegypti* (Aa) (AAW48724.1), *Drosophila melanogaster* (Dm) (NP_524453.1), *Acyrthosiphon pisum* (Acp) (XP_001944314.2), *Locusta migratoria* (Lm) (AFK29469.1), *Halyomorpha*

halys (Hh) (XP_014270680.1), *Bombyx mori* (Bm) (H9IX49), *Spodoptera litura* (Sl) (AHC98016.1), *Manduca sexta* (Ms) (JH668653.1).

Supplementary Figure S3b: Comparative domain architecture of Argonaute1 (Ago1) proteins: The domain in Ago1 proteins were predicted by ScanProsite, showed similar domain in all the above mentioned insects except in the case of *Acyrthosiphon pisum* and *Leptinotarsa decemlineata* where PIWI and PAZ domains are missing respectively, their Ago1 accession no. are *Agrilus planipennis*, Ap, AJF15704.1; *Tribolium castaneum*, Tc, A0A139WDH0; *Drosophila melanogaster*, Dm, NP_725341.1; *Acyrthosiphon pisum*, Acp, H8WGL9; *Aedes aegypti*, Aa, XP_001651170.1; *Manduca sexta*, Ms, JH668437.1; *Bombyx mori*, Bm, NP_001095931.1; *Spodoptera frugiperda*, Sf, AGS40929.1, *Spodoptera litura*, Sl, V9VID8; *Locusta migratoria*, Lm, AGO85968.1; *Leptinotarsa decemlineata*, Ld, LDEC011109-PA.

Supplementary Figure S3c: Comparative domain architecture of Argonaute3 (Ago3) proteins: The domain of Ago3 proteins were also predicted by ScanProsite, showed the presence of PIWI and PAZ domains different insects and their accession no. are *Bombyx mori*, Bm, NP_001098067.2; *Manduca sexta*, Ms, JH668363.1; *Tribolium castaneum*, Tc, XP_968053.2; *Leptinotarsa decemlineata*, Ld, LDEC003980-PA; *Acyrthosiphon pisum*, Acp, XP_003247838.2; *Spodoptera frugiperda*, Sf, AGS40929.1; *Agrilus planipennis*, Ap, APLA013701-PA; *Drosophila melanogaster,* Dm, ACJ13248.1; *Halyomorpha halys*, Hh, XP_014276831.1.

Supplementary Table 1: RNAi genes knockdown, and RNAi status of following insects

S. No.	Common Name	Scientific Name	Gene target	dsRNA degradation	siRNA equivalent band		RNAi status	References
					Injection	Feeding		
**COLEOPTERA**								
1	Colorado potato beetle	*Leptinotarsa decemlineata*	*Vacuolar ATPase subunits A* and *E*, Multiple targets	√	√	√	**Y**	1,2[F],3[FI],6
2	Spotted cucumber beetle	*Diabrotica undecimpunctata*	*Vacuolar ATPase subunits A* and *E*, *α- Tubulin*, *Snf7*, *A-Tubulin*	√	√	√	**Y**	1[F], 4[F], 5
3	Red flour beetle	*Tribolium castaneum*	*Vacuolar ATPase E*, *TcCHT5*, *TcCHT7*, *TcCHT10* and *TclDGF4*	√	√	√	**Y**	2,7,8,9
4	Emerald ash borer beetle	*Agrilus planipennis*	--	√	√	√	**Y**	10[I]
5	Eggplant flea beetle	*Epitrix fuscula*	--	√	√	√	**NI**	—
6	Japanese beetle	*Popillia japonica*	--	√	√	√	**NI**	—
7	Striped cucumber beetle	*Acalymma vittatum*	--	√	√	√	**NI**	—
8	Goldenrod soldier beetle	*Chauliognathus pensylvanicus*	--	√	√	√	**NI**	—
9	Lady bird beetle	*Coccinella septempunctata*	--	√	√	√	**NI**	—
10	Mexican bean beetle	*Epilachna varivestis*	--	√	√	√	**NI**	—
11	Pigweed flea beetle	*Disonycha glabrata*	--	√	√	√	**NI**	—
**LEPIDOPTERA**								
12	Codling moth	*Cydia pomonella*		–	**x**	–	**Y**	11[F]
13	Tobacco hornworm	*Manduca sexta*	*Vacuolar ATPase E*, Plasmatocyte-spreading peptide	√	**x**	–	**Y**	12[I], 7,13[I]
14	Cabbage looper	*Trichoplusia ni*		–	**x**	–	**NI**[#]	14
15	Fall armyworm	*Spodoptera frugiperda*	*Allatostatin C*, *Allatostatin 2*, *Cytochrome p450* (CYP6B1v4);*Trypsin SfT6*	√	**x**	**x**	**NI**[#]	15, 16, 17
16	Tobacco budworm	*Heliothis virescens*	--	√	**x**	**x**	**N**	3[FI]
17	Hairy caterpillar	*Spilosoma virginica*	--	√	**x**	–	**NI**	
18	Small purplish gray	*Iridopsis humaria*	--	–	**x**	–	**NI**	
19	Alfalfa caterpillar	*Colias eurytheme*	--	–	**x**	–	**NI**	
20	Saltmarsh caterpillar	*Estigmene acrea*	--	–	**x**	**x**	**NI**	

Supplementary Table 1: RNAi genes knockdown, and RNAi status of following insects

S. No.	Common Name	Scientific Name	Gene target	dsRNA degradation	siRNA equivalent band		RNAi status	References
					Injection	Feeding		
**HEMIPTERA**								
21	Whitefly	*Bemisia tabaci*		√	–	x	**Y**	18[I], 19[F]
22	Pea aphid	*Acyrthosiphon pisum*	*Vacuolar ATPase E*, *Water specific aquaporin*, *Hunchback*	√	x	x	**Y**	20[F], 21[I], 7, 22, 23, 24
23	Brown marmorated stink bug	*Halyomorpha halys*	--	√	√	x	**Y**	25[F]; 26[I],
24	Western tarnished plant bug	*Lygus hesperus*	--	√	√	–	**Y**	27[I]
25	Milk weed bug	*Oncopeltus fasciatus*	--	–	√	–	**Y**	28[I], 29[I]
26	Spined soldier bug	*Podisus maculiventris*	--	–	x	–	**NI**	
27	Milkweed assassin bug	*Zelus longipes*	--	–	x	–	**NI**	
28	Squash bug	*Anasa tristis*	--	√	√	x	**NI**	
29	Green stink bug	*Nezara viridula*	--	√	√	–	**NI**	
30	Harlequin bug	*Murgantia histrionica*	--	√	√	–	**NI**	
**DIPTERA**								
31	Mosquito	*Aedes aegypti*	*Vacuolar ATPase A*, Multi targets, *ATP-dependent efflux pump*	√	√	x	**Y**	30[F], 31[I] , 32, 33
32	Housefly	*Musca domestica*	--	√	√	–	**Y**	34[I], 35[I]
33	Fruit fly	*Drosophila melanogaster*	*Vacuolar ATPase E* and *Tubulin-γ*	√	x	x	**Y**	7, 36[I], 37[I]
34	Caribbean fruit fly	*Anastrepha suspensa*	--	√	x	x	**NI[##]**	38[I]
35	Syrphid fly	*Allograpta obliqua*	--	–	√	–	**NI**	
**ORTHOPTERA**								
36	Admirable grasshopper	*Syrbula admirabilis*	--	√	√	√	**NI**	
37	Field cricket	*Gryllus texensis*	--	√	√	–	**NI**	

√ = processed; X = not processed; – = not done
dsRNA processing experiments using injection only; RNAi workig (Y), Not working (N) and no information (NI)
No information for RNAi whole insect, however demosstrated in cell lines
No information for RNAi whole insect, however demosstrated through injecting the embryos
Superscript letter on references respresent: I-RNAi demonstrated through injection; F- RNAi demonstrated through Feeding;
FI- RNAi demonstrated through both Feeding and injection

References:

1 Baum, J. A. *et al.* Control of coleopteran insect pests through RNA interference. *Nature Biotechnology* **25**, 1322-1326 (2007).

2 Zhu, F., Xu, J., Palli, R., Ferguson, J. & Palli, S. R. Ingested RNA interference for managing the populations of the Colorado potato beetle, *Leptinotarsa decemlineata*. *Pest management science* **67**, 175-182 (2011).

3 Shukla, J. N. *et al.* Reduced stability and intracellular transport of dsRNA contribute to poor RNAi response in lepidopteran insects. *RNA biology* **13**, 656-669 (2016).

4 Levine, S. L. *et al.* Independent action between DvSnf7 RNA and Cry3Bb1 protein in southern corn rootworm, Diabrotica undecimpunctata howardi and Colorado potato beetle, *Leptinotarsa decemlineata*. *PloS One* **10**, e0118622 (2015).

5 Bolognesi, R. *et al.* Characterizing the Mechanism of Action of Double-Stranded RNA Activity against Western Corn Rootworm (*Diabrotica virgifera virgifera* LeConte). *PloS One* **7,** e47534 (2012).

6 Zhu, Q., Arakane, Y., Beeman, R. W., Kramer, K. J. & Muthukrishnan, S. Characterization of recombinant chitinase-like proteins of *Drosophila melanogaster* and *Tribolium castaneum*. *Insect biochemistry and molecular biology* **38**, 467-477 (2008).

7 Whyard, S., Singh, A. D. & Wong, S. Ingested double-stranded RNAs can act as species-specific insecticides. *Insect biochemistry and molecular biology* **39**, (2009).

8 Knorr, E., Bingsohn, L., Kanost, M. R. & Vilcinskas, A. in *Yellow Biotechnology II*163-178 (Springer, 2013).

9 Ulrich, J. *et al.* Large scale RNAi screen in *Tribolium* reveals novel target genes for pest control and the proteasome as prime target. *BMC genomics* **16**, 674 (2015).

10 Zhao, C., Gonzales, M. A. A., Poland, T. M. & Mittapalli, O. Core RNAi machinery and gene knockdown in the emerald ash borer (*Agrilus planipennis*). *Journal of insect physiology* **72**, 70-78 (2015).

11 Wang, K. *et al.* Variation in RNAi efficacy among insect species is attributable to dsRNA degradation in vivo. *Insect biochemistry and molecular biology* **77**, 1-9 (2016).

12 Eleftherianos, I. *et al.* Prior infection of *Manduca sexta* with non-pathogenic Escherichia coli elicits immunity to pathogenic *Photorhabdus luminescens*: roles of immune-related proteins shown by RNA interference. *Insect biochemistry and molecular biology* **36**, 517-525 (2006).

13 Garbutt, J. S., Belles, X., Richards, E. H. & Reynolds, S. E. Persistence of double-stranded RNA in insect hemolymph as a potential determiner of RNA interference success: evidence from *Manduca sexta* and *Blattella germanica*. *Journal of insect physiology* **59**, 171-178 (2013).

14 Kim, N. Y. *et al.* Short-hairpin RNA-mediated gene expression interference in *Trichoplusia ni* cells. *Journal of microbiology and biotechnology* **22**, 190-198 (2012).

15 Griebler, M., Westerlund, S. A., Hoffmann, K. H. & Meyering-Vos, M. RNA interference with the allatoregulating neuropeptide genes from the fall armyworm *Spodoptera frugiperda* and its effects on the JH titer in the hemolymph. *Journal of insect physiology* **54**, 997-1007 (2008).

16 Rodriguez-Cabrera, L., Trujillo-Bacallao, D., Borras-Hidalgo, O., Wright, D. J. & Ayra-Pardo, C. RNAi-mediated knockdown of *a Spodoptera frugiperda* trypsin-like serine-protease gene reduces susceptibility to a *Bacillus thuringiensis* Cry1Ca1 protoxin. *Environmental microbiology* **12**, 2894-2903 (2010).

17 Ghosh, S. *et al.* RNAi Screening in *Spodoptera frugiperda*. *High-Throughput RNAi Screening: Methods and Protocols*, 199-212 (2016).

18 Ghanim, M., Kontsedalov, S. & Czosnek, H. Tissue-specific gene silencing by RNA interference in the whitefly Bemisia tabaci (Gennadius). *Insect biochemistry and molecular biology* **37**, 732-738 (2007).

19 Upadhyay, S. K. *et al.* siRNA Machinery in Whitefly (*Bemisia tabaci*). *PloS One* **8**, e83692 (2014).

20 Mutti, N. S., Park, Y., Reese, J. C. & Reeck, G. R. RNAi knockdown of a salivary transcript leading to lethality in the pea aphid, *Acyrthosiphon pisum*. *Journal of insect science* **6**, 1-7 (2006).

21 Jaubert-Possamai, S. *et al.* Gene knockdown by RNAi in the pea aphid *Acyrthosiphon pisum*. *BMC Biotechnology* **7**, 63 (2007).

22 Shakesby, A. J. *et al.* A water-specific aquaporin involved in aphid osmoregulation. *Insect biochemistry and molecular biology* **39**, 1-10 (2009).

23 Mao, J. & Zeng, F. Feeding-Based RNA Intereference of a Gap Gene Is Lethal to the Pea Aphid, *Acyrthosiphon pisum*. *PloS One* **7**, e48718 (2012).
24 Christiaens, O., Swevers, L. & Smagghe, G. DsRNA degradation in the pea aphid (*Acyrthosiphon pisum*) associated with lack of response in RNAi feeding and injection assay. *Peptides* **53**, 307-314 (2014).
25 Ghosh, S. K. B., Hunter, W. B., Park, A. L. & Gundersen-Rindal, D. E. Double strand RNA delivery system for plant-sap-feeding insects. *PloS One* **12**, e0171861 (2017).
26 Bansal, R. & Michel, A. P. Core RNAi Machinery and Sid1, a Component for Systemic RNAi, in the Hemipteran Insect, *Aphis glycines*. *International Journal of Molecular Sciences* **14**, 3786-3801 (2013).
27 Van Ekert, E., Wang, M., Miao, Y. G., Brent, C. & Hull, J. RNA interference-mediated knockdown of the Halloween gene Spookiest (CYP307B1) impedes adult eclosion in the western tarnished plant bug, *Lygus hesperus*. *Insect molecular biology* **25**, 550-565 (2016).
28 Hrycaj, S., Mihajlovic, M., Mahfooz, N., Couso, J. P. & Popadić, A. RNAi analysis of nubbin embryonic functions in a hemimetabolous insect, *Oncopeltus fasciatus*. *Evolution & development* **10**, 705-716 (2008).
29 Hughes, C. L. & Kaufman, T. C. RNAi analysis of Deformed, proboscipedia and Sex combs reduced in the milkweed bug *Oncopeltus fasciatus*: novel roles for Hox genes in the hemipteran head. *Development* **127**, 3683-3694 (2000).
30 Coy, M. R. *et al.* Gene silencing in adult *Aedes aegypti* mosquitoes through oral delivery of double-stranded RNA. *Journal of Applied Entomology* **136**, 741-748 (2012).
31 Drake, L. L., Price, D. P., Aguirre, S. E. & Hansen, I. A. RNAi-mediated gene knockdown and in vivo diuresis assay in adult female *Aedes aegypti* mosquitoes. *JoVE (Journal of Visualized Experiments)*, e3479-e3479 (2012).
32 Figueira-Mansur, J. *et al.* Silencing of P-glycoprotein increases mortality in temephos-treated *Aedes aegypti* larvae. *Insect molecular biology* **22**, 648-658 (2013).
33 Singh, A. D., Wong, S., Ryan, C. P. & Whyard, S. Oral Delivery of Double-Stranded RNA in Larvae of the Yellow Fever Mosquito, *Aedes aegypti*: Implications for Pest Mosquito Control. *Journal of Insect Science* **13**, 69 (2013).
34 Gao, Y. *et al.* Downregulation of the *Musca domestica* peptidoglycan recognition protein SC (PGRP-SC) leads to overexpression of antimicrobial peptides and tardy pupation. *Molecular immunology* **67**, 465-474 (2015).
35 Siegenthaler, C., Maroy, P., Hediger, M., Dübendorfer, A. & Bopp, D. Hormones and sex-specific transcription factors jointly control yolk protein synthesis in *Musca domestica*. *International journal of evolutionary biology* **2009,** (2009).
36 Dzitoyeva, S., Dimitrijevic, N. & Manev, H. Intra-abdominal injection of double-stranded RNA into anesthetized adult *Drosophila* triggers RNA interference in the central nervous system. *Molecular psychiatry* **6**, 665 (2001).
37 Dzitoyeva, S., Dimitrijevic, N. & Manev, H. γ-aminobutyric acid B receptor 1 mediates behavior-impairing actions of alcohol in *Drosophila*: adult RNA interference and pharmacological evidence. *Proceedings of the National Academy of Sciences* **100**, 5485-5490 (2003).
38 Schetelig, M. F., Milano, A., Saccone, G. & Handler, A. M. Male only progeny in *Anastrepha suspensa* by RNAi-induced sex reversion of chromosomal females. *Insect biochemistry and molecular biology* **42**, 51-57 (2012).

Supplementary Table 2: List of insects, place of collection and treatment methods

S. No	Common name/ stage of insect/ Number of insect used for feeding, injection	Scientific name	Place/ Host of collection	Feeding	Injection
1	Colorado potato beetle/ Grub/ (2,2)	*Leptinotarsa decemlineata*	Potato/ Culture maintained in green house	5 µl of dsGFP sandwiched between two leaf disc punched with eppendorf tube	5 µl of dsGFP injected with insulin syringe
2	Spotted cucumber beetle/ Adult/ (5,5)/	*Diabrotica undecimpunctata*	Cucumber/ UKY South farm, Lexington, KY	5 µl of dsGFP sandwiched between two leaf disc of cucumber punched with eppendorf tube	325nl/insect injected with Nanoject II (Durmond Scientific Inc)
3	Striped cucumber beetle/ Adult/ (5,5)	*Acalymma vittatum*	Cucumber/ UKY South farm, Lexington, KY	5 µl of dsGFP sandwiched between two leaf disc of cucumber punched with eppendorf tube	325nl/insect injected with Nanoject II (Durmond Scientific Inc)
4	Eggplant Flea Beetle/ Adult/ (50,20)	*Epitrix fuscula*	Egg plant/ UKY South farm, Lexington, KY	5 µl of dsGFP sandwiched between two leaf disc of egg plant punched with eppendorf tube	325nl/insect injected with Nanoject II (Durmond Scientific Inc)
5	Japanese beetle / Grub/(2,2)	*Popillia japonica*	Insect culture from UKY Entomology Department	1.5 mm cube of carrot soaked with 5 µl dsGFP s	5 µl of dsGFP injected with insulin syringe
6	Red flour beetle/ Grub (20,10)	*Tribolium castaneum*	Insect culture from UKY Entomology Department	Forced feeding 325 nl dsGFP directed into the oral cavity of 4th instar grub	325nl/insect injected with Nanoject II (Durmond Scientific Inc)

7	Ladybird beetle/ Adult (3,3)	*Coccinella septempunctata*	Insect culture from UKY Entomology Department	Fed with Aphids which were fed on artificial diet (30 % sucrose + 5 % Yeast) incorporated with 5 µl dsGFP	5 µl of dsGFP injected with insulin syringe
8	Mexican bean beetle (4)	*Epilachna varivestis*	Cucumber/ UKY South farm, Lexington, KY	—	5 µl of dsGFP injected with insulin syringe
9	Emerald ash borer beetle (4, 2)	*Agrilus planipennis*	UKY Entomology Department	Fed on artificial diet (30 % sucrose + food colour dye) incorporated with 5 µl dsGFP	5 µl of dsGFP injected with insulin syringe
10	Goldenrod soldier beetle (4)	*Chauliognathus pensylvanicus*	Fennel plant/ UKY South farm, Lexington, KY	—	5 µl of dsGFP injected with insulin syringe
11	Pigweed flea beetle (4)	*Disonycha glabrata*	UKY South farm, Lexington, KY	—	5 µl of dsGFP injected with insulin syringe
**Lepidoptera**					
1	Codling moth/ Larvae (2)	*Cydia pomonella*	Apple Culture maintained in green house	—	5 µl of dsGFP injected with insulin syringe
2	Hairy caterpillar/ Larvae (2)	*Spilosoma virginica*	Tobacco/ UKY North farm, Lexington, KY	—	"
3	Small Purplish Gray / Larvae (2)	*Iridopsis humaria*	Soyabean/ UKY North farm, Lexington, KY	—	"
4	Cabbage Looper/ Larvae (2)	*Trichoplusia ni*	Soyabean/ UKY North farm, Lexington, KY	—	"

5	Alfalfa caterpillar/ Larvae (2)	*Colias eurytheme*	Soyabean/ UKY North farm, Lexington, KY	—	"
6	Tobacco hornworm/ Larvae (2)	*Manduca sexta*	Tobacco/ UKY North farm, Lexington, KY	—	"
7	Saltmarsh caterpillar/ Larvae (2)	*Estigmene acrea*	Insect culture maintained at UKY Entomology Department	3mm cube noctuid diet soaked with 5 µl of dsGFP	"
8	Tobacco budworm/ Larvae (2)	*Heliothis virescens*	Insect culture from UKY Entomology Department	3mm cube noctuid diet soaked with 5 µl of dsGFP	"
9	Fall armyworm/Larvae (2)	*Spodoptera furgiperda*	Insect culture from UKY Entomology Department	3mm cube noctuid diet soaked with 5 µl of dsGFP	"
**Hemiptera**					
**1**	Whitefly (100 adults)	*Bemisia tabaci*	Insect culture from UKY Entomology Department	Artificial diet (30 % sucrose + 5 % Yeast) incorporated with 5 µl dsGFP placed between two layers of par film	—
2	Pea aphid (10, 20)	*Acyrthosiphon pisum*	Insect culture from UKY Entomology Department	Artificial diet (30 % sucrose + 5 % Yeast) incorporated with 5 µl dsGFP placed between two layers of parafilm	—

3	Brown marmorated stink bug/ Adult (2,2)	*Halyomorpha halys*	Insect culture from UKY Entomology Department	5 mm piece of fresh green bean soaked with 5 µl of dsGFP	5 µl of dsGFP injected with insulin syringe
4	Squash bug/ Adult (2,2)	*Anasa tristis*	Squash/ UKY South farm, Lexington, KY	5 mm piece of fresh squash soaked with 5 µl of dsGFP	5 µl of dsGFP injected with insulin syringe
5	Green stink bug/ Adult (2)	*Nezara viridula*	Soyabean/ UKY North farm, Lexington, KY	—	5 µl of dsGFP injected with insulin syringe
6	Milkweed bug /Adult (2)	*Oncopeltus fasciatus*	Squash/ UKY North farm, Lexington, KY	—	325nl/insect injected with Nanoject II (Durmond Scientific Inc)
7	Spined soldier bug /Adult (2)	*Podisus maculiventris*	Cucumber/ UKY North farm, Lexington, KY	—	325nl/insect injected with Nanoject II (Durmond Scientific Inc)
8	Milkweed assassin bug / Adult (2)	*Zelus longipes*	Squash/ UKY North farm, Lexington, KY	—	325nl/insect injected with Nanoject II (Durmond Scientific Inc)
9	Western tarnished plant bug/ Adult (2)	*Lygus hesperus*	Squash/ UKY North farm, Lexington, KY	—	325nl/insect injected with Nanoject II (Durmond Scientific Inc)

10	Harlequin bug	*Murgantia histrionica*	Sun flower/Arboretum, UKY, Lexington, KY	—	5 µl of dsGFP injected with insulin syringe
**Diptera**					
1	Mosquito/ Adults (20,10)	*Aedes aegypti*	Insect culture from UKY Entomology Department	Fed with artificial diet (30 % sucrose) incorporated with 5 µl dsGFP	325nl/insect injected with Nanoject II (Durmond Scientific Inc)
2	Syrphid fly/ Grub (2)	*Allograpta obliqua*	Cucumber/ UKY North farm, Lexington, KY	—	325nl/insect injected with Nanoject II (Durmond Scientific Inc)
3	Housefly/ Adults (10)	*Musca domestica*	From SC Johnson, Wisconsin	—	325nl/insect injected with Nanoject II (Durmond Scientific Inc)
4	Fruit fly	*Drosophila melanogaster*	Insect culture from UKY Entomology Department	—	2 µl of dsGFP injected with insulin syringe
5	Caribbean fruit fly	*Anastrepha suspensa*	Insect culture from UKY Entomology Department	—	5 µl of dsGFP injected with insulin syringe
**Mecoptera**					
1	Scorpion fly/ Adults (5)	*Panorpa confusa*	Soybean/ UKY North farm, Lexington, KY	—	325nl/insect injected with Nanoject II (Durmond Scientific Inc)
**Collembollan**					

1	Collembola/ Adults (100)		Insect culture from UKY Entomology Department	Soaked with 20 ml water + 5 µl dsGFP	—
**Isoptera**					
1	Termite/ Adults (20,10)	*Reticulitermes flavipes*	Insect culture from UKY Entomology Department	5 µl of dsGFP sandwiched between two tissue paper disc punched with eppendorf tube	325nl/insect injected with Nanoject II (Durmond Scientific Inc)
**Orthoptera**					
1	Horse head grasshopper/ Adults(2,2)	*Syrbula admirabilis*	Squash/ UKY North farm, Lexington, KY	5 µl of dsGFP sandwiched between two leaf disc of beans punched with eppendorf tube	5 µl of dsGFP injected with insulin syringe
2	Field Cricket	*Gryllus texensis*	Insect culture from UKY Entomology Department	—	5 µl of dsGFP injected with insulin syringe
**Hymenoptera**					
1	Ponerine Ant/ Adults (3)	*Hypoponera opacior*	Insect culture from UKY Entomology Department	Fed 30 % sucrose incorporated with 5 µl dsGFP	—
2	Pavement ant/ Adults (3)	*Tetramorium caespitum*	Insect culture from UKY Entomology Department	Fed 30 % sucrose incorporated with 5 µl dsGFP	—

The numbers in parentheses after common name of insect are the insect numbers selected

Supplementary Figure S1a: (A)

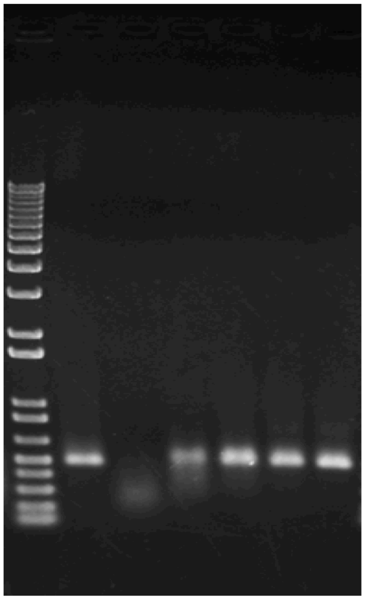

Popillia japonica **(Pj)**

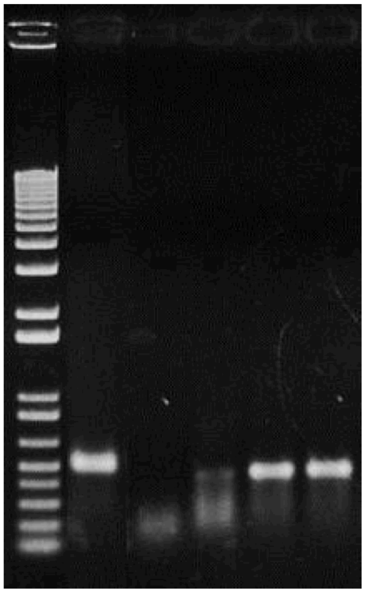

Epilachna varivestis **(Ev)**

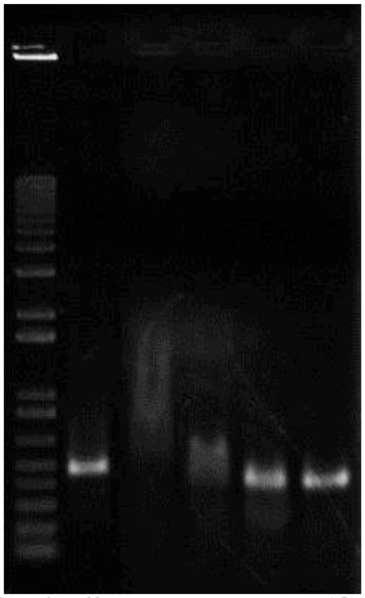

Coccinella septempunctata **(Cs)**

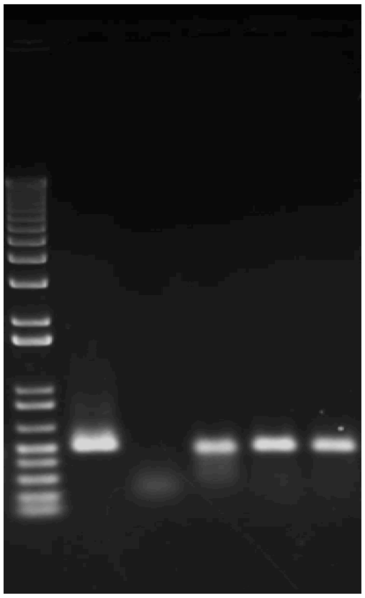

Disonycha glabrata **(Dg)**

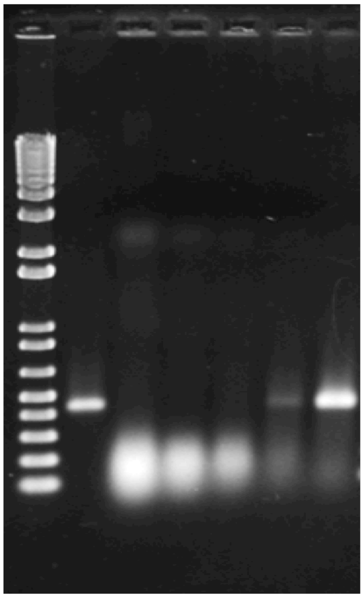

Leptinotarsa decemlineata **(Ld)**

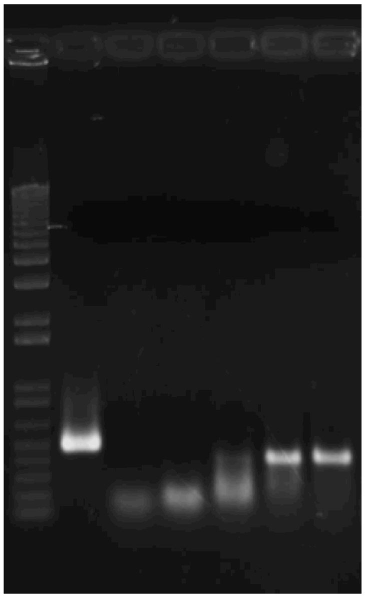

Acalymma vittatum **(Av)**

Supplementary Figure S1a: (B)

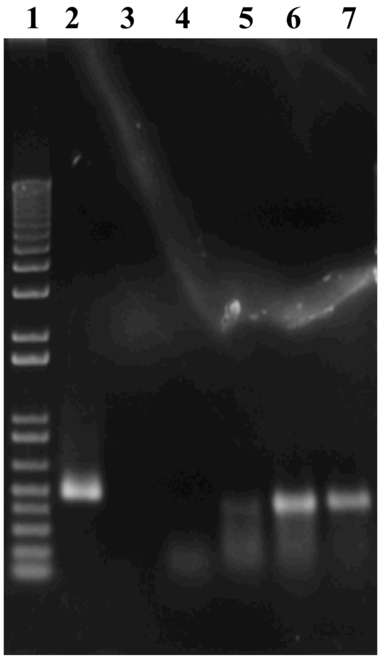

Epitrix fuscula (Ef)

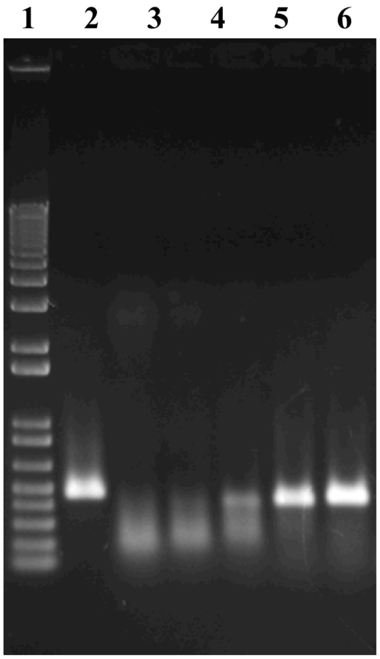

Diabrotica undecimpunctata (Du)

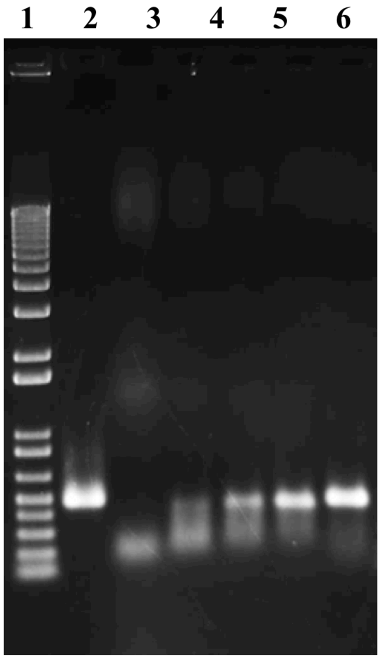

Chauliognathus pensylvanicus (Cp)

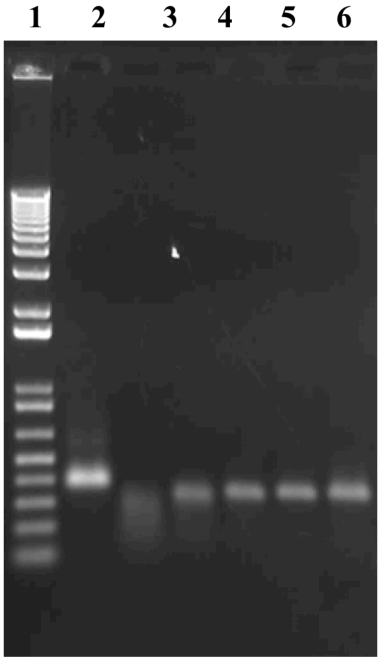

Tribolium castaneum (Tc)

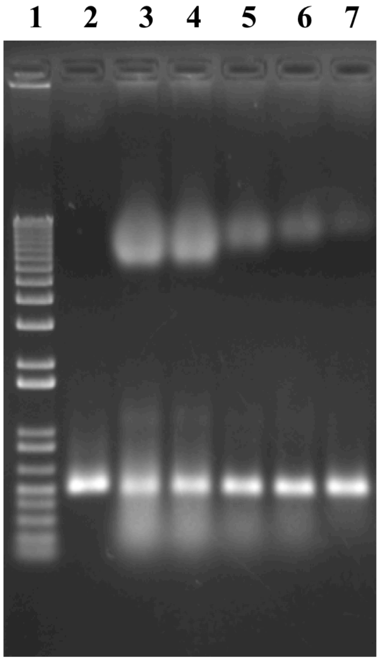

Agrilus planipennis (Ap)

Supplementary Figure S1c:

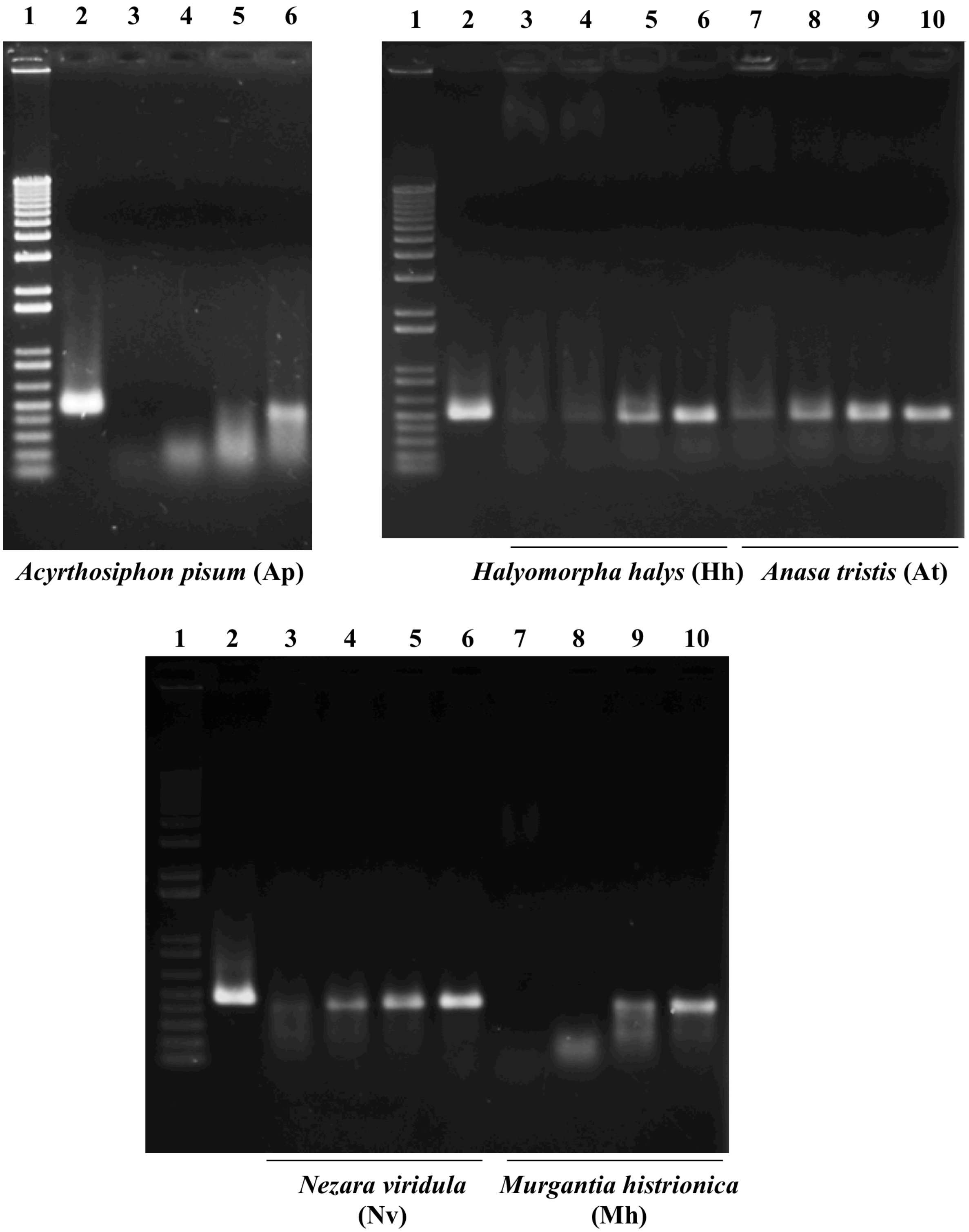

Supplementary Figure S1d:

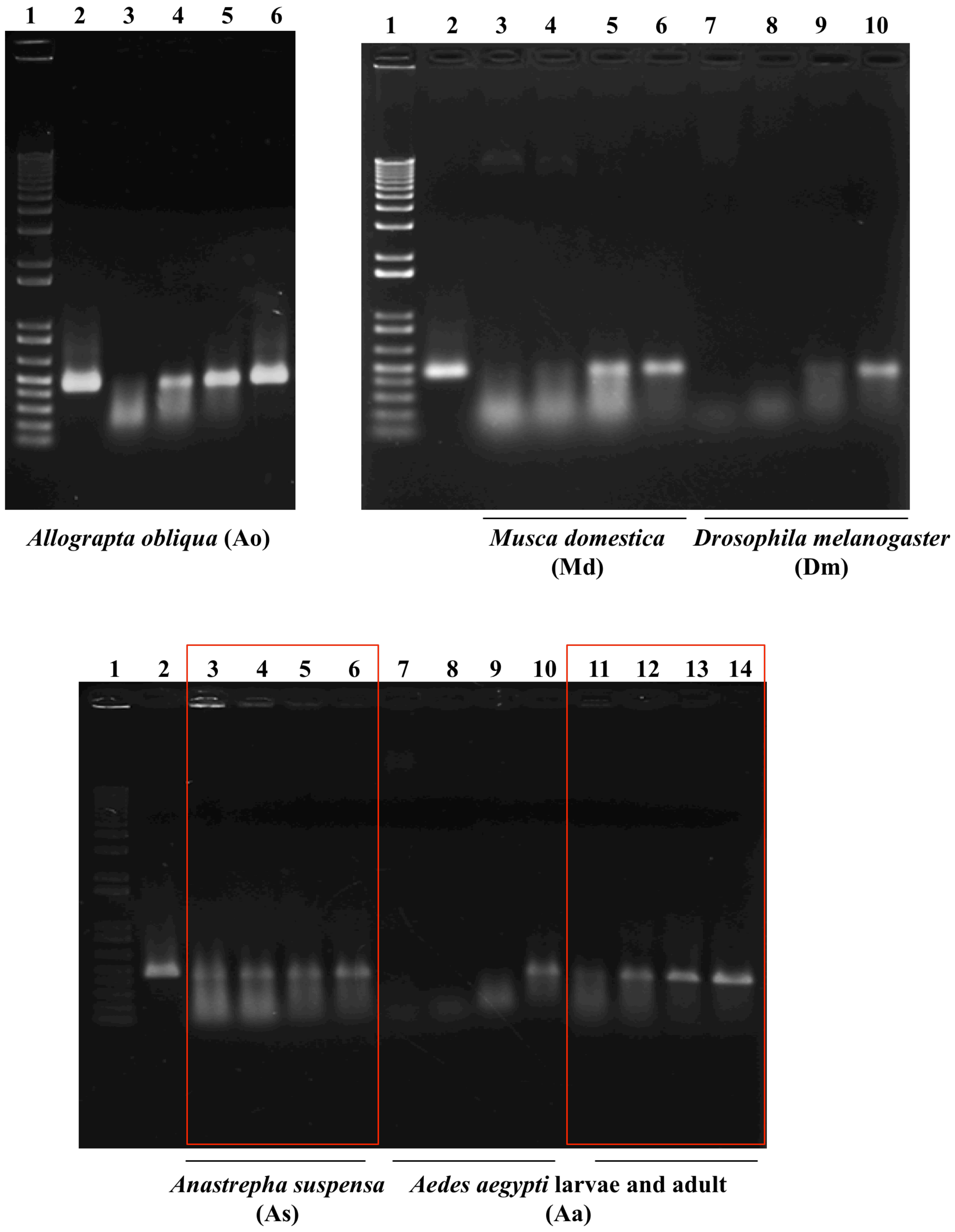

Supplementary Figure S1e:

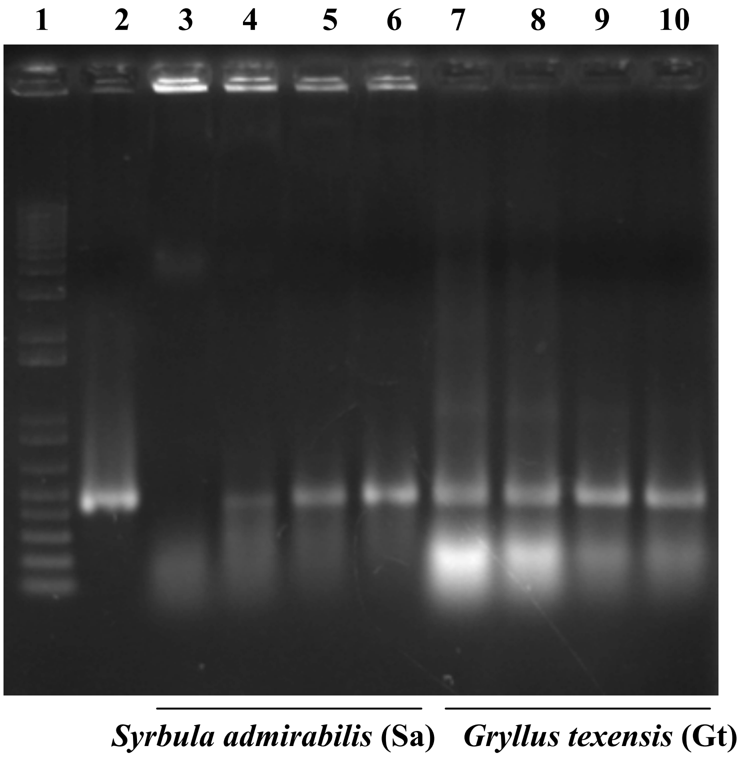

Supplementary Figure S2a:

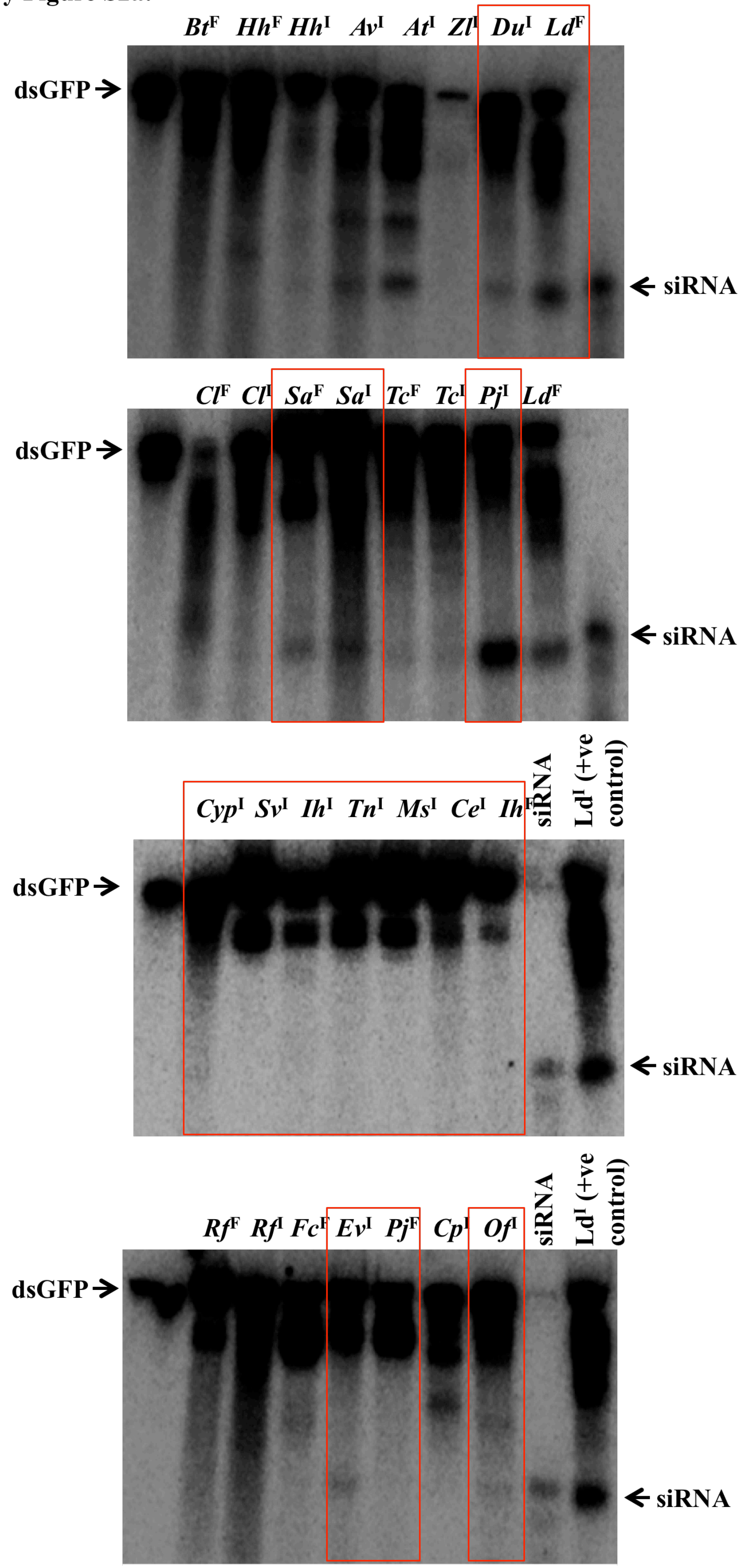

Supplementary Figure S2b:

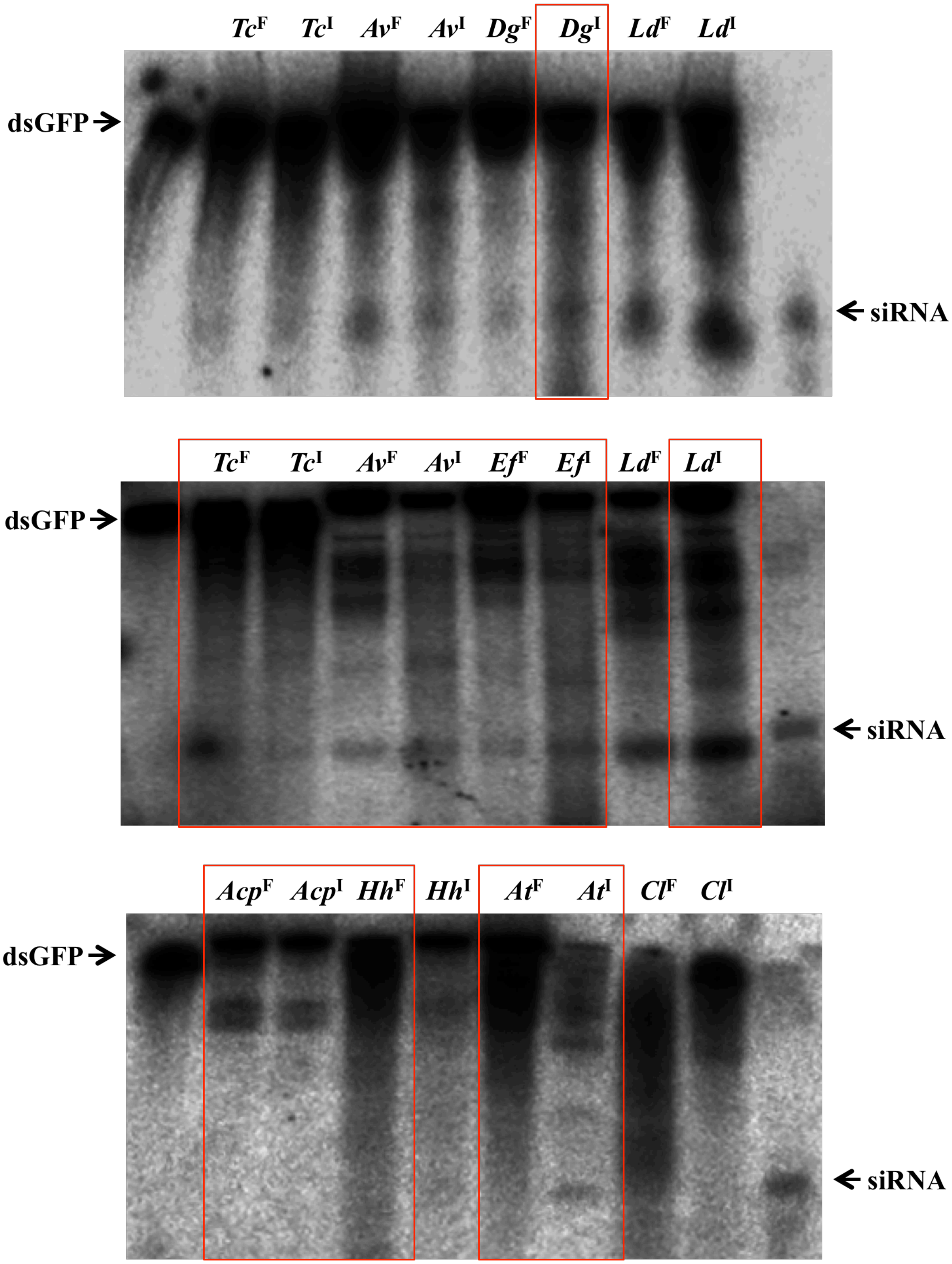

Supplementary Figure S2c:

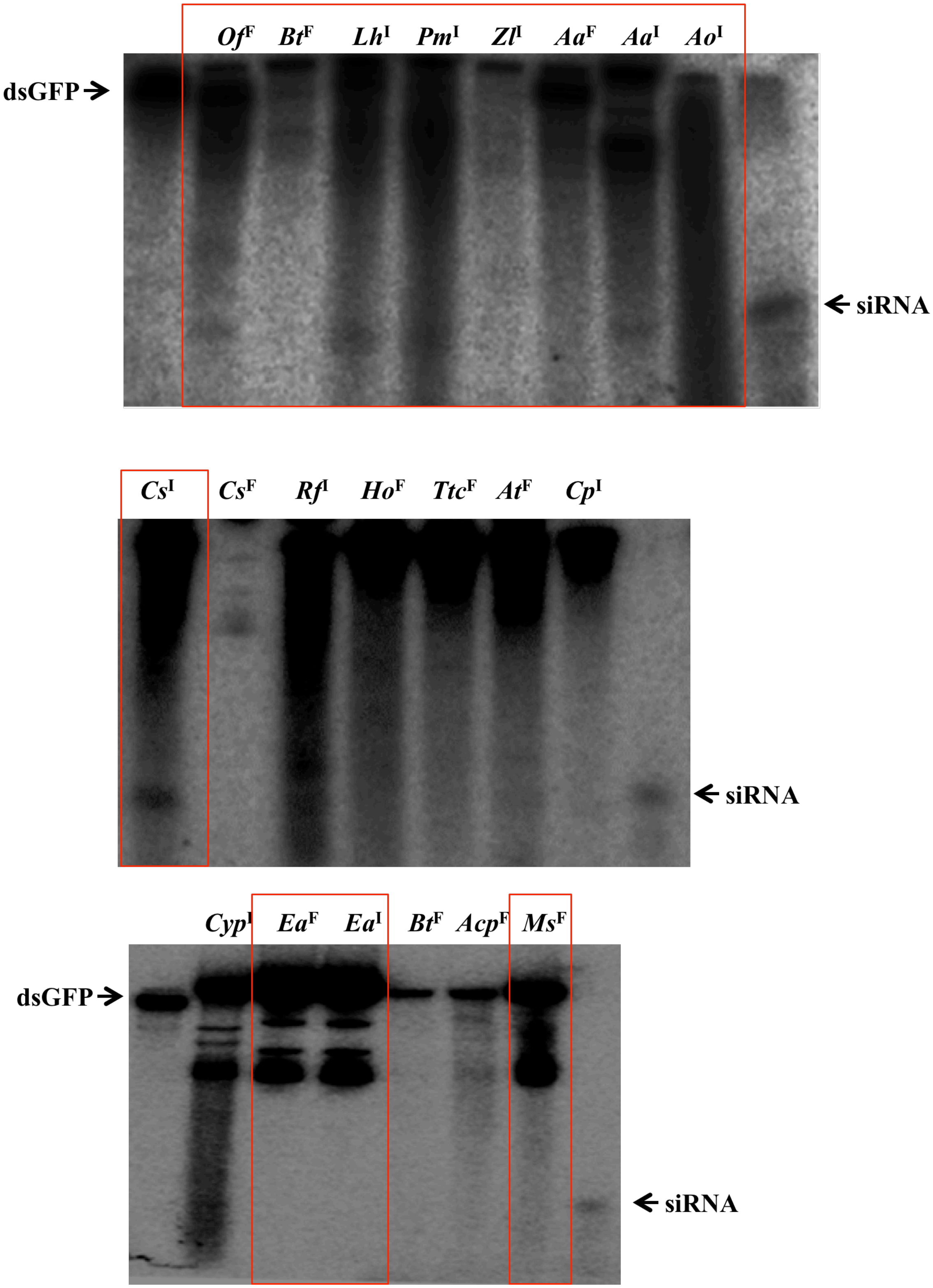

Supplementary Figure S2d:

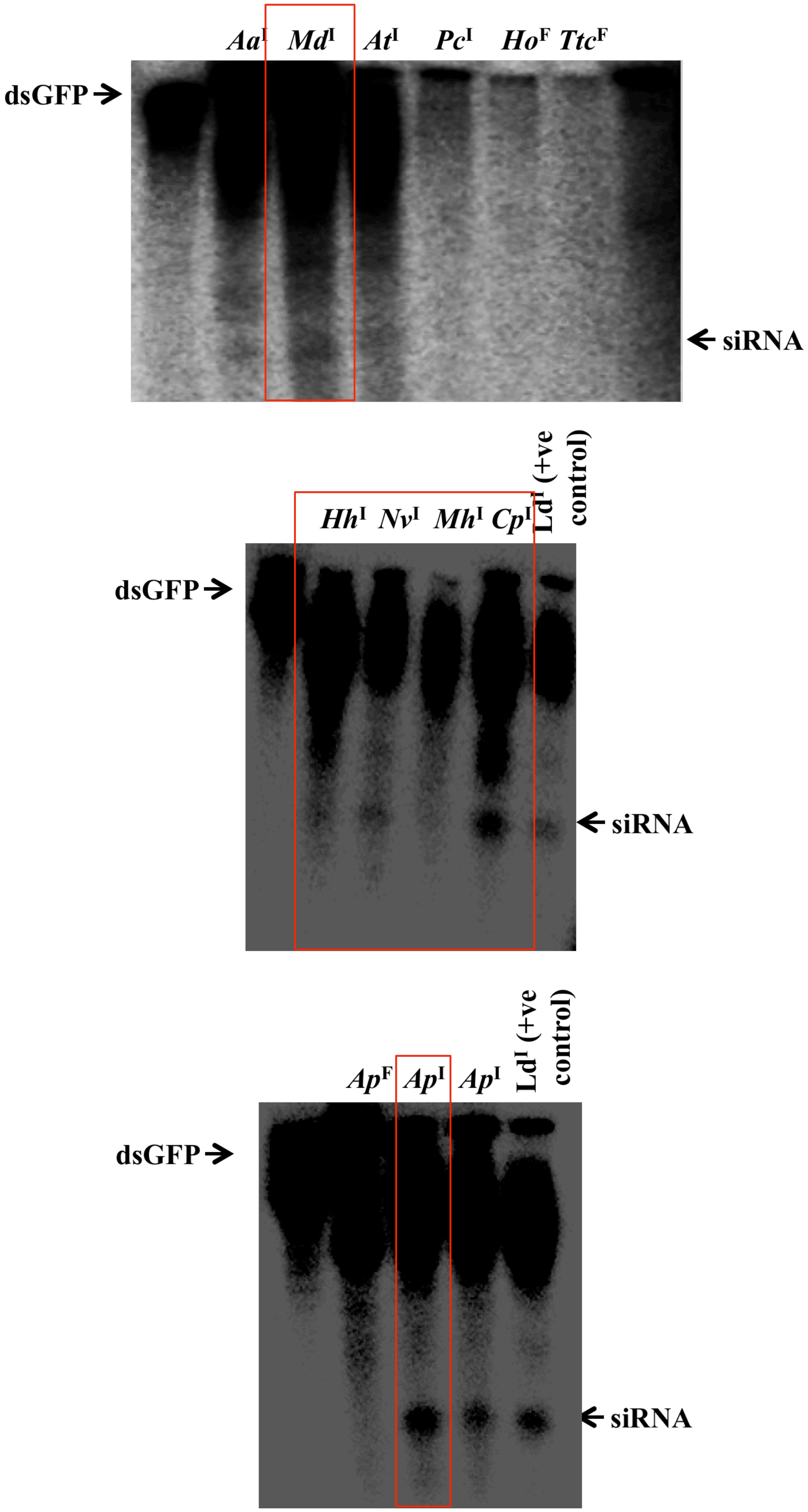

Supplementary Figure S2e:

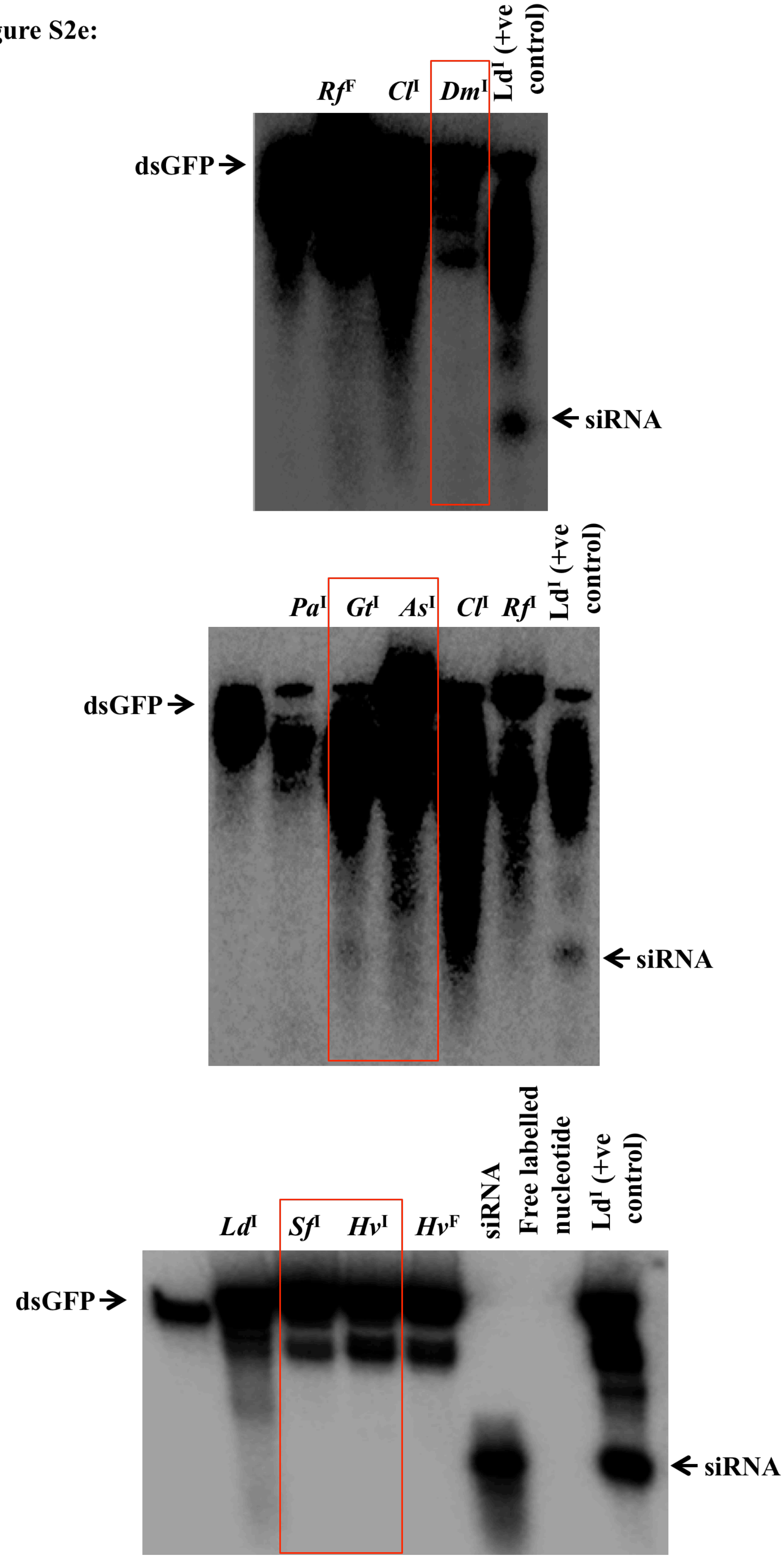

Supplementary Figure S3a

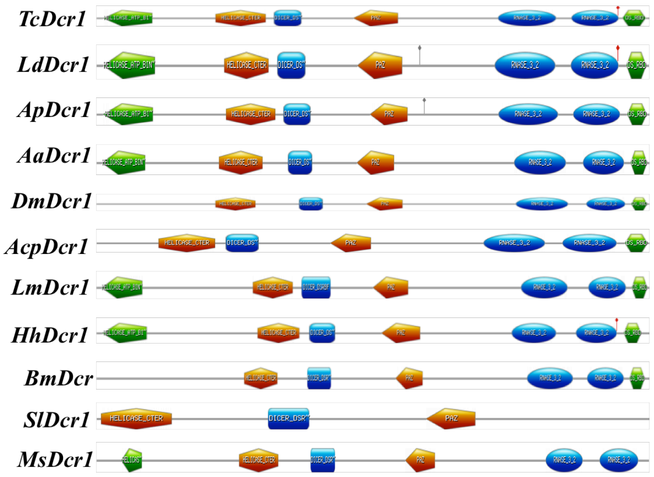

Supplementary Figure S3b

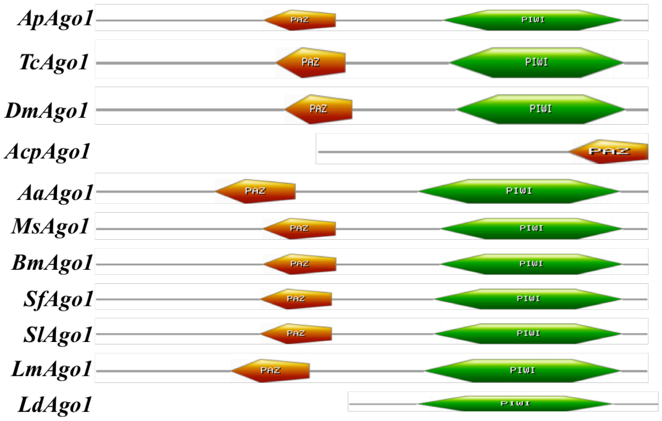

Supplementary Figure S3c

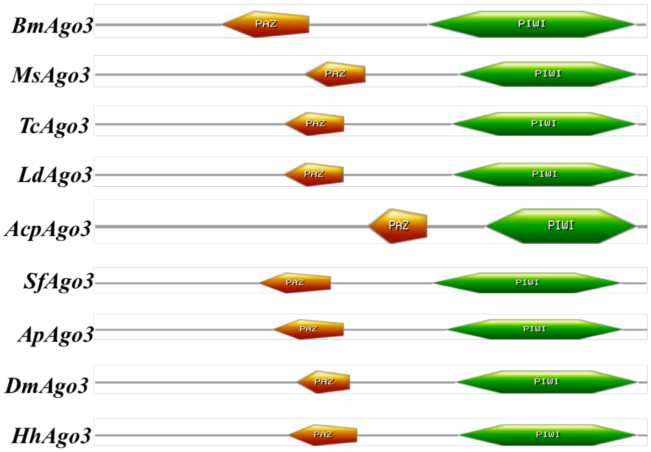

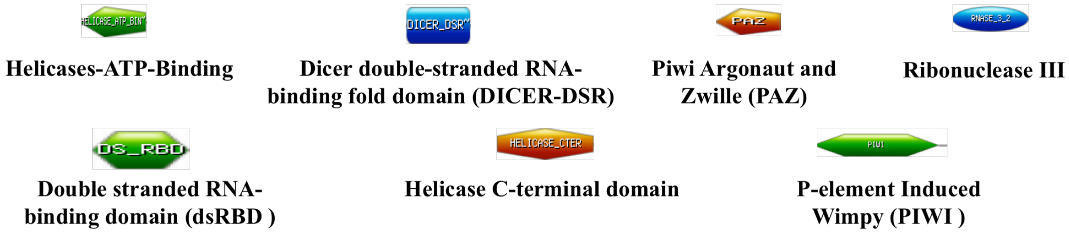

Supplement: Supplementary file 1 — Supplementary Information [file 41598_2017_17134_MOESM1_ESM.pdf]
